# Coordination Polymer Flexibility Leads to Polymorphism and Enables a Crystalline Solid–Vapour Reaction: A Multi-technique Mechanistic Study

**DOI:** 10.1002/chem.201500514

**Published:** 2015-05-11

**Authors:** Iñigo J Vitórica-Yrezábal, Stefano Libri, Jason R Loader, Guillermo Mínguez Espallargas, Michael Hippler, Ashleigh J Fletcher, Stephen P Thompson, John E Warren, Daniele Musumeci, Michael D Ward, Lee Brammer

**Affiliations:** [a] Department of Chemistry, University of Sheffield Brook Hill, Sheffield S3 7HF (UK) Fax: (+44) 114-222-9536 E-mail: lee.brammer@sheffield.ac.uk Homepage: http://www.sheffield.ac.uk/chemistry/staff/profiles/lee_brammer; [b] Instituto de Ciencia Molecular (ICMol), Universidad de Valencia c/Catedrático José Beltrán 2, 46980 Paterna (Spain); [c] Department of Chemical and Process Engineering, University of Strathclyde 75 Montrose St, Glasgow G1 1XJ (Scotland); [d] Diamond Light Source, Harwell Science and Innovation Campus Didcot, Oxon OX11 0DE (UK); [e] School of Materials, University of Manchester Manchester M13 9PL (UK); [f] Molecular Design Institute, NYU Department of Chemistry 100 Washington Square East, New York, NY 10003 (USA)

**Keywords:** coordination polymers, gas-phase spectroscopy, in situ diffraction, microscopy, polymorphism, porosity, solid-state reactions, thermal analysis

## Abstract

Despite an absence of conventional porosity, the 1D coordination polymer [Ag_4_(O_2_C(CF_2_)_2_CF_3_)_4_(TMP)_3_] (**1**; TMP=tetramethylpyrazine) can absorb small alcohols from the vapour phase, which insert into Ag–O bonds to yield coordination polymers [Ag_4_(O_2_C(CF_2_)_2_CF_3_)_4_(TMP)_3_(ROH)_2_] (**1-ROH**; R=Me, Et, *i*Pr). The reactions are reversible single-crystal-to-single-crystal transformations. Vapour-solid equilibria have been examined by gas-phase IR spectroscopy (*K*=5.68(9)×10^−5^ (MeOH), 9.5(3)×10^−6^ (EtOH), 6.14(5)×10^−5^ (*i*PrOH) at 295 K, 1 bar). Thermal analyses (TGA, DSC) have enabled quantitative comparison of two-step reactions **1-ROH**→**1**→**2**, in which **2** is the 2D coordination polymer [Ag_4_(O_2_C(CF_2_)_2_CF_3_)_4_(TMP)_2_] formed by loss of TMP ligands exclusively from singly-bridging sites. Four polymorphic forms of **1** (**1-A^LT^**, **1-A^HT^**, **1-B^LT^** and **1-B^HT^**; HT=high temperature, LT=low temperature) have been identified crystallographically. In situ powder X-ray diffraction (PXRD) studies of the **1-ROH**→**1**→**2** transformations indicate the role of the HT polymorphs in these reactions. The structural relationship between polymorphs, involving changes in conformation of perfluoroalkyl chains and a change in orientation of entire polymers (A versus B forms), suggests a mechanism for the observed reactions and a pathway for guest transport within the fluorous layers. Consistent with this pathway, optical microscopy and AFM studies on single crystals of **1-MeOH**/**1-A^HT^** show that cracks parallel to the layers of interdigitated perfluoroalkyl chains develop during the MeOH release/uptake process.

## Introduction

The study of coordination polymers now spans some 25 years.[1] In their porous form, as metal–organic frameworks, close to 20 years of research[2] has produced a substantial variety of materials with wide-ranging properties, including gas storage and separation,[3] catalysis,[4] medical applications,[5] magnetic,[6] electronic[7] and optical properties.[8] There has been increasing recognition in recent years that despite being periodic coordinate covalent assemblies, these materials are far from static in the solid state and can undergo substantial structural and chemical changes, often while retaining crystallinity, either as a polycrystalline material or in some cases as a single crystal.[9] Kole and Vittal have recently reviewed such transformations in coordination polymers, describing a wide range of phenomena, including solvent removal/uptake, changes in network dimensionality, photochemical reactions, mechanochemical reactions, gas-solid reactions, ligand addition/removal, and ligand or metal ion replacement.[10] In contrast to the level of mechanistic understanding in solution-phase molecular chemistry, however, little is known about the mechanism in most cases of such transformations in solid-state coordination polymers. This is partially due to the novelty of these transformations as well as to the experimental and computational challenges in monitoring or modelling such transformations in the solid state.

Uptake of molecules from the gas or vapour phase by non-porous crystalline materials remains rare, although the number of examples of this phenomenon is growing, typically owing to crystalline architectures that permit molecular motions in crystals, which give rise to dynamic (or transient) porosity.[11, 12] In some cases the uptake/release of small molecules also involves the formation or cleavage of covalent bonds.[13–17] Previously, we reported investigations focused on reactions of vapours or gases with non-porous crystalline molecular compounds and coordination polymers that involve metal–ligand bond breaking/formation in the solid state.[14, 16]

**Scheme 1:**
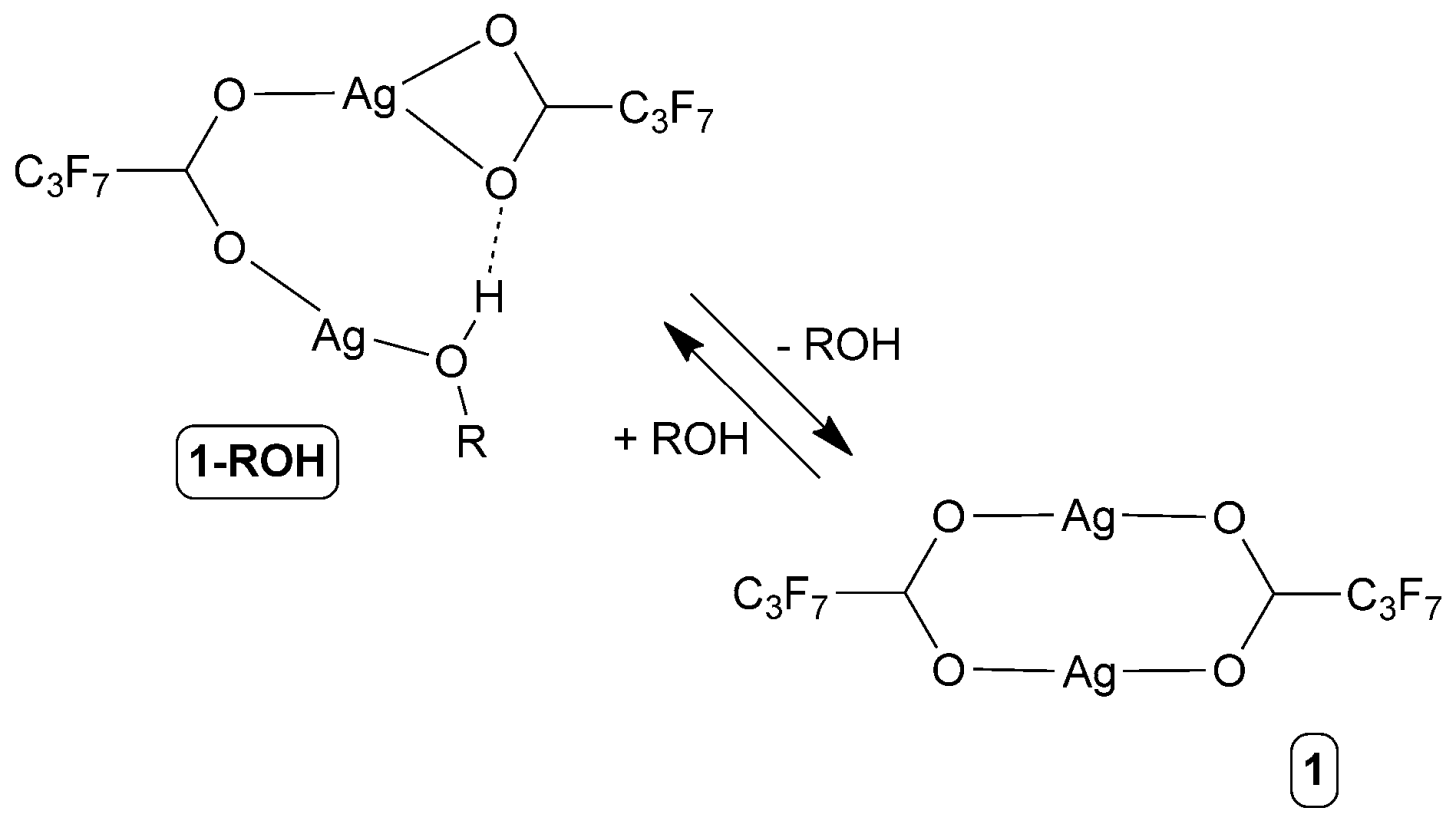
Reversible alcohol release/uptake by the coordination polymers [Ag_4_(O_2_C(CF_2_)_2_CF_3_)_4_(TMP)_3_(ROH)_2_] (1-ROH; R=Me, Et, *i*Pr) yielding coordination polymer 1, here emphasising only the change in carboxylate coordination.

**Scheme 2:**
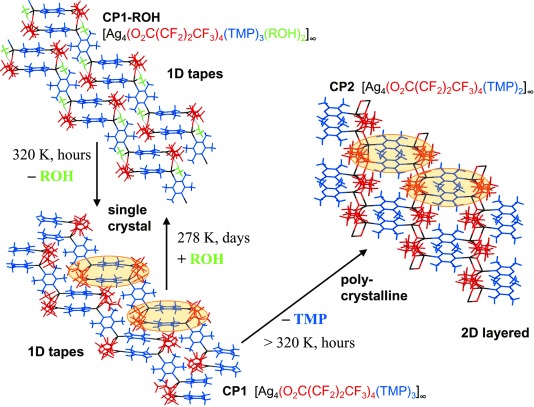
Reaction scheme showing the relationship between coordination polymers 1-ROH (shown here with R=Me), 1 (as polymorph 1-A^HT^) and 2.

Our laboratory demonstrated that single crystals of the one-dimensional (1D) coordination polymer [Ag_4_(O_2_C(CF_2_)_2_CF_3_)_4_(TMP)_3_] (**1**; TMP=2,3,5,6 tetramethylpyrazine), which exhibits a non-porous architecture in which the polymer chains are aligned with each other and interact through interdigitated perfluoroalkyl chains extending from the carboxylate ligands, undergoes reversible uptake and release of small alcohols in their vapour phase.[16b] These single-crystal-to-single-crystal transformations (SCSCTs) involve insertion/deinsertion of the alcohol molecules into metal–carboxylate (Ag–O) bonds, accompanied by formation or breaking, respectively, of O–H⋅⋅⋅O hydrogen bonds between the coordinated alcohol molecules and a carboxylate group (Scheme 1). Uptake of small alcohols (ROH) generates coordination polymer **1-ROH**. Coordination polymer **1** also can release one-third of its TMP ligands into the vapour phase, resulting in an irreversible transformation to the 2D layered coordination polymer [Ag_4_(O_2_C(CF_2_)_2_CF_3_)_4_(TMP)_2_] (**2**). The reaction manifold is summarised in Scheme 2. Herein we describe a mechanistic investigation of the solid-state reaction pathways using multiple techniques, which has led to the discovery that **1** is polymorphic (four phases, **1-A^LT^**, **1-A^HT^**, **1-B^LT^** and **1-B^HT^**; HT=high temperature, LT=low temperature). Moreover, this investigation reveals that interconversion between the polymorphs can be attributed to the structural flexibility of the coordination polymers, enabling guest transport and TMP release (**1**→**2**).

## Results

### Crystal structures and the solid–vapour reaction manifold

**Figure 1:**
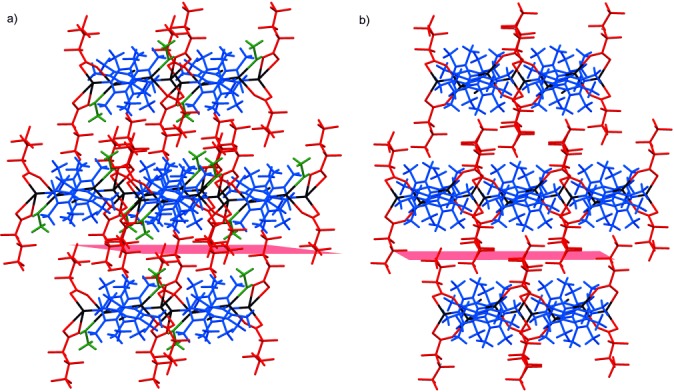
View of a) 1-MeOH and b) 1-A^HT^ along the coordination polymer chains, illustrating the rod-like distorted hexagonal packing motif of these chains. The coordination polymer propagation direction is approximately [113] for 1-MeOH and is [001] for 1-A^HT^. The (01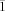) planes for 1-MeOH and (010) planes for 1-A^HT^ (shown in red) lie parallel to the fluorous layers. Ag in black, TMP ligands in blue, heptafluorobutanoate in red, methanol in green. Note that lattice indexing is different for the two crystal structures. Alternative views of these structures are shown in Figures 7 b and 8 b.

The previously reported crystal structures [Ag_4_(O_2_C(CF_2_)_2_CF_3_)_4_(TMP)_3_(MeOH)_2_] (**1-MeOH**), [Ag_4_(O_2_C(CF_2_)_2_CF_3_)_4_(TMP)_3_(EtOH)_2_] (**1-EtOH**) and [Ag_4_(O_2_C(CF_2_)_2_CF_3_)_4_(TMP)_3_(*i*PrOH)_2_] (**1-*i*PrOH**) are isostructural with the example denoted as **1-ROH** in Scheme 2. These coordination polymers comprise pairs of Ag^I^ cations that are bridged directly by a heptafluorobutanoate ligand. A second heptafluorobutanoate ligand adopts an asymmetric coordination mode in which it forms a chelate with one Ag centre and a hydrogen bond (O–H⋅⋅⋅O) with an alcohol molecule coordinated to the other Ag centre (Scheme 1). The di-silver units, Ag_2_(O_2_C(CF_2_)_2_CF_3_)_2_(ROH) (R=Me, Et, or *i*Pr), are linked by pairs of parallel TMP ligands. The resulting tetra-silver units, Ag_4_(O_2_C(CF_2_)_2_CF_3_)_4_(TMP)_2_(ROH)_2_, are linked further through single TMP ligands to form a polymeric zigzag tape. The one-dimensional coordination polymers **1-ROH** propagate approximately in the [113] direction and assemble in a distorted hexagonal rod-like packing motif such that each polymer tape is surrounded by six neighbouring tapes (Figure 1 a). Perfluoroalkyl groups of neighbouring polymers are interdigitated to provide fluorous layers that lie parallel to the (01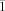) planes. The coordinated alcohol is lost upon mild heating of **1-ROH** (R=Me, Et and *i*Pr) coordination polymers, yielding the coordination polymer [Ag_4_(O_2_C(CF_2_)_2_CF_3_)_4_(TMP)_3_] (**1**) (Scheme 2), which adopts four different polymorphic forms (vide infra), each with crystal structures closely related to that of **1-ROH**. The transformation from **1-ROH** to **1** requires the breaking of Ag–O(H)R coordination bonds and RO–H⋅⋅⋅O_carboxylate_ hydrogen bonds and the formation of new Ag–O_carboxylate_ bonds, that is, effectively an intramolecular ligand substitution reaction at alternate Ag centres. Further heating of **1** leads to loss of the singly- bridging TMP ligands and formation of new Ag–O bonds that directly link the remaining tetra-silver units, Ag_4_(O_2_C(CF_2_)_2_CF_3_)_4_(TMP)_2_, into the more condensed 2D coordination polymer **2** (Scheme 2).

### Thermal gravimetric analysis/differential scanning calorimetry (TGA-DSC)

A study of **1-MeOH**, **1-EtOH** and **1-*i*PrOH** by combined TGA-DSC clearly shows sequential loss of two alcohol molecules (step 1) followed by one TMP ligand (step 2) per [Ag_4_(O_2_C(CF_2_)_2_CF_3_)_4_(TMP)_3_(ROH)_2_] formula unit (Table 1; Figure S26 in the Supporting Information), consistent with the previously established structural characterisation for reaction of **1-MeOH**.[16b] These events precede a final larger mass loss (step 3) in which remaining organic components of the materials are removed. At the scan rate used (2 °C min^−1^) loss of TMP is completed within the temperature range 383–458 K in each case. EtOH loss is complete at a higher temperature (383 K) than MeOH loss (348 K). *i*PrOH loss is complete at 348 K, a lower temperature than for EtOH loss. This is consistent with difference in preparation of **1-*i*PrOH** (from solid–vapour reaction of **1** and *i*PrOH) compared to **1-MeOH** and **1-EtOH** (solution-phase synthesis), as discussed previously.[16b] The identities of the products formed after each step were confirmed as [Ag_4_(O_2_C(CF_2_)_2_CF_3_)_4_(TMP)_3_] **(1)** and [Ag_4_(O_2_C(CF_2_)_2_CF_3_)_4_(TMP)_2_] **(2)**, respectively, by independent powder and/or single-crystal X-ray diffraction experiments. The data for the final decomposition step are consistent with formation of a mixture of Ag_2_O and Ag_2_CO_3_.

**Table 1:** TGA-DSC results for heating of compounds 1-MeOH, 1-EtOH and 1-*i*PrOH

		1-MeOH(25.925 mg)	1-EtOH(26.025 mg)	1-*i*PrOH(25.2397 mg)
step 1 (loss of ROH)	temp range			
	TGA:	303–348 K	328–383 K	333–348 K
	DSC:	321–359 K	345–392 K	339–355 K
	mass loss (%)	expected 3.66	expected 5.16	expected 6.63
		observed 3.43	observed 5.15	observed 6.40
	enthalpy [kJ mol^−1^]	77.77	67.31	98.11
				
step 2 (loss of TMP)	temp range			
	TGA:	398–456 K	383–438 K	393–441 K
	DSC:	415–458 K	409–436 K	402–436 K
	mass loss [%]	expected 7.44	expected 7.12	expected 7.52
		observed 7.77	observed 7.64	observed 7.51
	enthalpy [kJ mol^−1^]	50.76	51.31	52.90
				
step 3 (decomp)	temp range			
	TGA:	480–507 K	483–509 K	480–508 K
	DSC:	483–507 K	484–511 K	484–510 K
	final mass [%]	observed 29.63	observed 28.46	observed 28.86
		expected (Ag_2_O): 26.45	expected (Ag_2_O): 25.97	expected (Ag_2_O): 25.57
		expected (Ag_2_CO_3_): 31.47	expected (Ag_2_CO_3_): 30.91	expected (Ag_2_CO_3_): 30.43

### Polymorphs of 1

**Figure 2:**
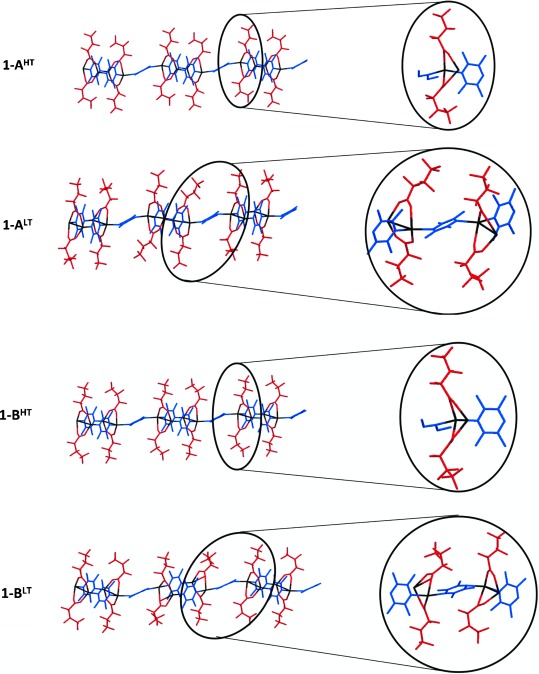
Crystal structures of the four polymorphs (1-A^HT^, 1-A^LT^, 1-B^HT^ and 1-B^LT^) of coordination polymer 1 [Ag_4_(O_2_C(CF_2_)_2_CF_3_)_4_(TMP)_3_]. Expansions show the asymmetric units, highlighting: i) the coordination environment of the Ag^I^ centres; and ii) the different conformational distributions (*gauche*:*anti)* of the perfluoroalkyl chains. Hydrogen atoms are not shown. In expanded views TMP ligands are only shown in part for polymorphs 1-A^HT^ and 1-B^HT^. Colour code as in Figure 1.

Four polymorphs have been identified and crystallographically characterised for coordination polymer **1**. Each of two structure types (**A** and **B**) adopts a high temperature (HT) and low temperature (LT) form (**1-A^LT^**, **1-A^HT^**, **1-B^LT^** and **1-B^HT^**). Polymorph **1-A^HT^** adopts the crystal structure previously reported by our laboratory as **1**.[16b] Polymorph **1-B^HT^** was previously reported as **1^HT^**, based on unit cell dimensions alone, determined from powder diffraction in our earlier report (i.e., no crystal structure determination). The four polymorphs share a common polymeric zig-zag tape motif in which pairs of Ag^I^ cations adopt a dimeric arrangement bridged by two carboxylate ligands, these dimers being propagated into the tape by the TMP ligands through alternating double and single bridges. The A and B polymorph structures differ primarily with respect to the arrangement of the coordination polymer tapes, whereas the difference between LT and HT forms stems from the conformations of the fluoroalkyl groups and a reduction in symmetry in the LT forms (Figure 2).

**Figure 3:**
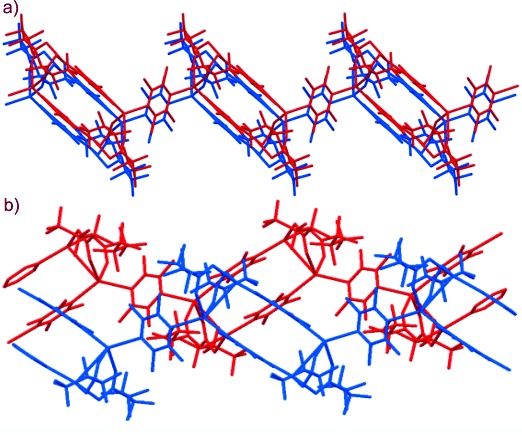
a) Crystal structure of polymorph 1-A^HT^ viewed along the [010] direction showing tapes stacked directly upon each other and b) crystal structure of polymorph 1-B^HT^ viewed along the [001] direction showing tapes that are related by a 2_1_ screw axis that lies along the vertical axis of this view (i.e. [010]). Blue and red colours denote adjacent tapes stacked along the viewing direction. Hydrogen atoms not shown.

Interdigitated perfluoroalkyl groups of neighbouring polymers provide fluorous layers in the crystal (e.g., see Figure 1 b for **1-A^HT^**), as previously noted for **1-ROH**. The fluoroalkyl groups exhibit some disorder towards the end of the chain (away from the carboxylate group), suggesting mobility of these groups, possibly as a result of the relatively weak dispersion interactions. The perfluoroalkyl groups adopt one of two conformations (*gauche* or *anti*) about their C_β_–C_γ_ bond. The LT forms (**1-A^LT^** and **1-B^LT^**) have four crystallographically independent perfluorocarboxylate groups, which in polymorph **1-A^LT^** exist in a 3:1 ratio of *anti*:*gauche* conformations, whereas for polymorph **1-B^LT^** a 1:1 *anti*:*gauche* ratio is observed. The HT polymorphs (**1-A^HT^** and **1-B^HT^**) each have only two crystallographically independent perfluorocarboxylate groups, present in a 1:1 ratio of *anti*:*gauche* conformations. Polymorph **1-A^LT^** has been observed in the temperature range of 100–115 K (temperatures below 100 K have not been examined) and reversibly forms polymorph **1-A^HT^** at about 115 K. Polymorph **1-A^HT^** has been observed in the temperature range of 115–340 K, and forms polymorph **1-B^HT^** at approximately 340 K. Polymorph **1-B^HT^** has been observed in the temperature range of 250–340 K and reversibly forms **1-B^LT^** at about 250 K. In polymorphs **1-A^HT^** and **1-A^LT^** polymeric tapes are stacked along the [010] and [001] directions, respectively, and are related by inversion symmetry, such that Ag_4_(O_2_C(CF_2_)_2_CF_3_)_4_(TMP)_2_ units in adjacent tapes have parallel orientations (Figure 3 a). In polymorphs **1-B^HT^** and **1-B^LT^**, tapes are related by a 2_1_ screw axis parallel to the *b* axis, such that Ag_4_(O_2_C(CF_2_)_2_CF_3_)_4_(TMP)_2_ units in adjacent tapes are rotated by approximately 72° (Figure 3 b). These arrangements are of consequence in the mechanism proposed in the Discussion section.

### In situ powder diffraction studies of solid-state reactions involving loss of coordinated alcohol: 1-EtOH→1 and 1-*i*PrOH→1

**Figure 4:**
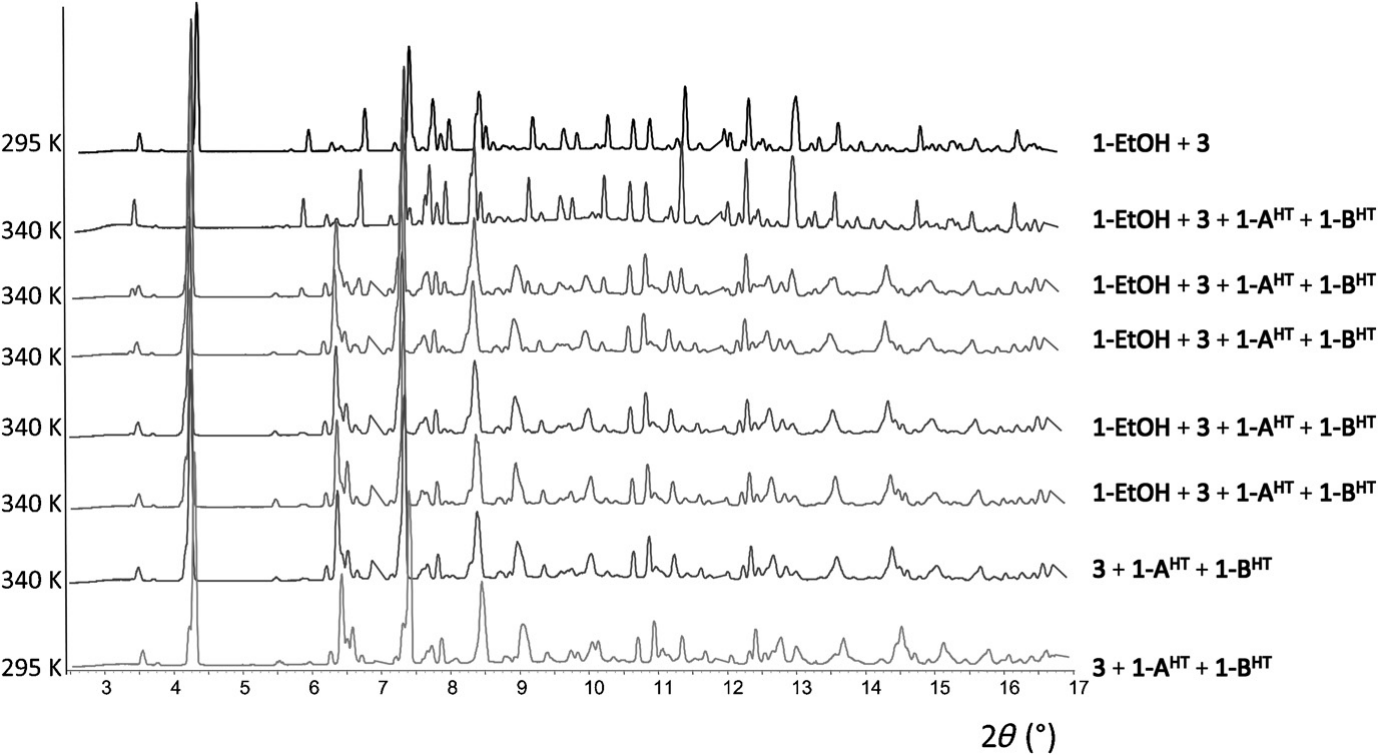
In situ synchrotron powder diffraction study of ethanol release reaction, 1-EtOH→1-A^HT^+1-B^HT^, at 340 K (from top to bottom). Interval between powder patterns is 20 min. (Compound 3 is an impurity phase that remains unchanged during the conversion of 1-EtOH to 1). (Rietveld fits are shown in Figures S7–S14 in the Supporting Information).

**Figure 5:**
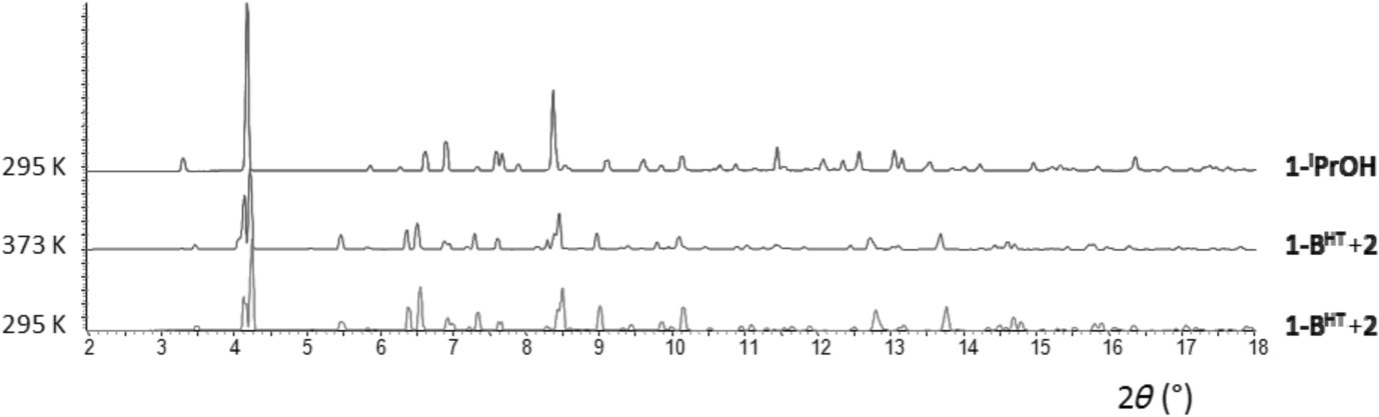
In situ synchrotron powder diffraction study of *i*PrOH release reaction, 1-*i*PrOH→1-B^HT^+2, at 373 K (from top to bottom). Interval between powder patterns is 20 min. (Rietveld fits are shown in Figures S15–S17 in the Supporting Information).

The release of ethanol and isopropanol vapours from crystalline **1-EtOH** (Figure 4) and **1-*i*PrOH** (Figure 5), respectively, was monitored in situ using synchrotron X-ray powder diffraction at 340 K for compound **1-EtOH** and at 373 K for compound **1-*i*PrOH**. Patterns were collected every 20 min and Rietveld refinement[18] was used to fit the diffraction pattern at each time interval and determine the proportion of each constituent present. An impurity phase (**3**, 25 %) was found in the sample of compound **1-EtOH**, but the contribution of **3** to the diffraction pattern is unchanged upon heating the sample.[19] Formation of compound **1** was observed after 20 min of heating for each compound. Two different polymorphs, **1-A^HT^** and **1-B^HT^**, were observed on heating compound **1-EtOH**. After 2 h of heating, release of ethanol by **1-EtOH** was complete (Figure 4). Only polymorph **1-B^HT^** was observed upon isopropanol release by **1-*i*PrOH**, although it is possible that **1-A^HT^** was formed and converted to **1-B^HT^** between measurement steps. The release of isopropanol by **1-*i*PrOH** was completed after 20 min (Figure 5) and traces of compound **2** were also detected, indicating some subsequent loss of the TMP ligand from **1** as previously documented by in situ diffraction for **1-MeOH**[16b] and confirmed herein by TGA-DSC for all **1-ROH** compounds.

### Gas-phase infrared spectroscopy

In order to establish that the reactions conform to a solid–vapour equilibrium process, the partial pressure of alcohol vapour was monitored by gas-phase FT-IR spectroscopy during its release from crystalline **1-ROH**, using a previously devised procedure[14b] that employs a custom-built gas-phase IR cell (Figure S19 in the Supporting Information).

**Figure 6:**
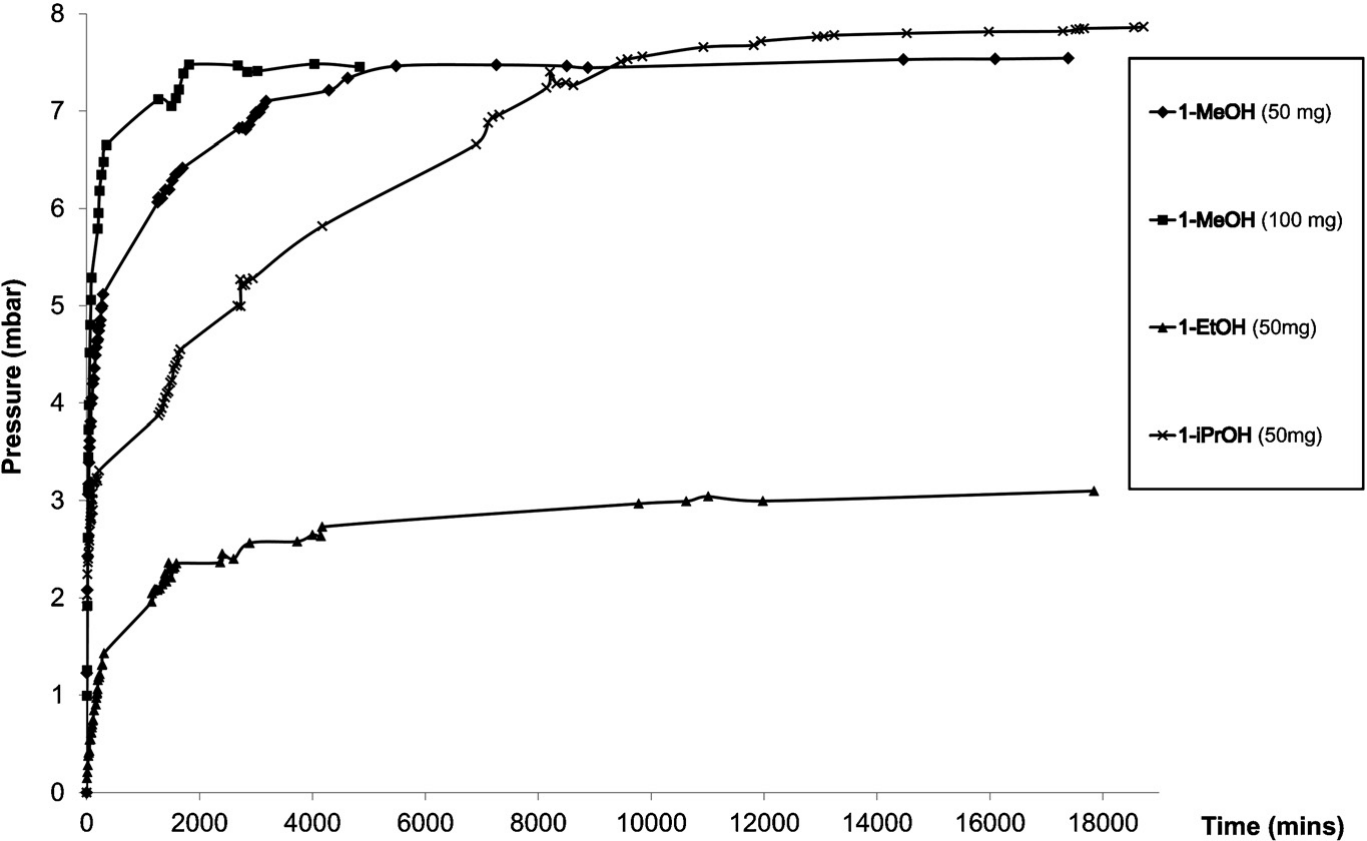
Evolution of the partial pressure of MeOH, EtOH and *i*PrOH with time.

The alcohol release reactions reach equilibrium [Eq. (1)] after several days, at which time the equilibrium pressure could be determined (Figure 6). Table 2 lists the equilibrium constant, *K*_p_ (at 295 K; *p*^o^=1 bar), and the corresponding Δ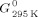 for each of the three alcohols. Equilibrium constants were calculated by assuming unit fugacity coefficients for all vapours at the low pressures involved.[20] The validity of using unit activity coefficients for the crystalline solids was examined and confirmed by conducting measurements with different quantities of **1-MeOH** (Table 2, Figure 6).

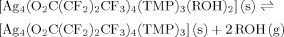
(1)

**Table 2:** Final partial pressure, equilibrium constant (K_p_ at 295 K, *p*^o^=1 bar) and Gibbs free energy for alcohol release reactions at 295 K

	Final partial pressure of ROH^[a]^ [bar]	*K*_p_	Δ 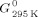 [kJ mol^−1^]^[a]^
**1-MeOH** (50 mg)	0.00753(6)	5.68(9)×10^−5^	24.22(4)
**1-MeOH** (100 mg)	0.00744(5)	5.55(7)×10^−5^	24.27(3)
**1-EtOH**	0.00308(5)	9.5(3)×10^−6^	28.65(8)
**1-*i*PrOH**	0.00783(2)	6.14(5)×10^−5^	24.02(1)

[a] Errors are determined by from the spread of values obtained once the pressure has reached a plateau. The average of these values is the reported pressure.

### Optical and atomic force microscopy

In order to investigate whether alcohol loss and uptake takes place through particular crystal faces, the loss of MeOH from **1-MeOH** to form **1-A^HT^** and the uptake of MeOH by **1-A^HT^** to form **1-MeOH** was examined by optical microscopy and AFM using single crystals.

**Figure 7:**
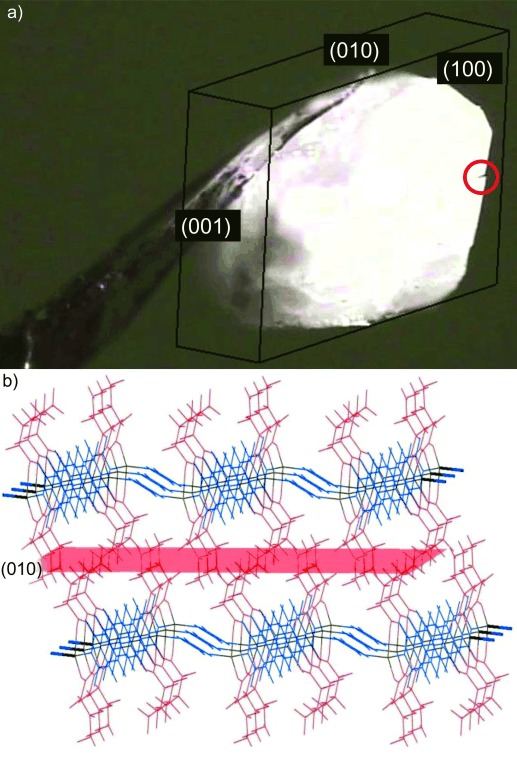
a) Single crystal of 1-A^HT^ (formed by MeOH loss from 1-MeOH) with crystal faces indicated, showing a small crack (circled), which lies parallel to the (010) plane (indexing relative to 1-A^HT^ lattice). b) Crystal structure of 1-A^HT^ view of the perpendicular to the (100) plane with (010) plane shown in red. Colours are as in Scheme 2. Hydrogen atoms not shown.

A single crystal of **1-MeOH** mounted on an X-ray diffractometer was allowed to lose MeOH at room temperature under a dry nitrogen stream, during which time the unit cell of the crystal was monitored by X-ray diffraction and optical images of the crystal were recorded using a CCD microscope. After 2.5 h, unit cell determinations confirmed that complete conversion of **1-MeOH** to **1-A^HT^** had occurred. An image of the crystal viewed approximately perpendicular to the large (100) face of **1-A^HT^** indicates that a small crack developed parallel to the (010) planes of **1-A^HT^** (Figure 7 a). The planes correspond to the fluorous regions that result from interdigitated fluoroalkyl groups of neighbouring coordination polymers (Figure 7 b).

**Figure 8:**
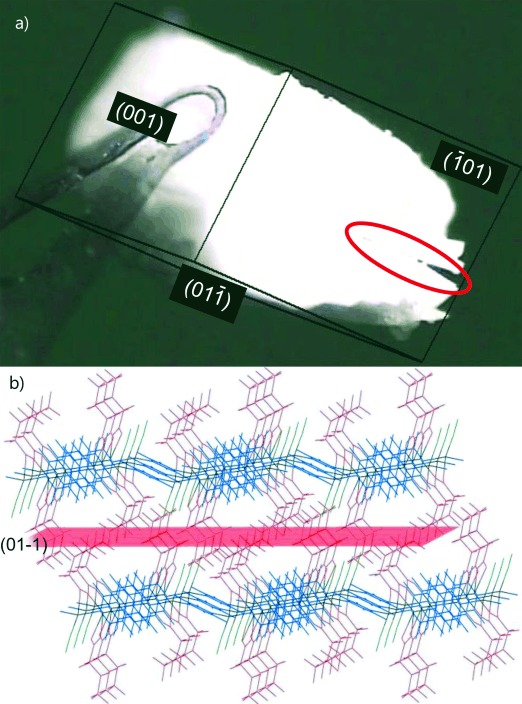
a) Single crystal of crystal 1-MeOH, formed from crystal of 1-A^HT^ after 24 h exposure to MeOH vapour, indicated the enlarged crack (circled), which lies parallel to the (01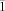) planes (indexing relative to 1-MeOH lattice). b) Crystal structure of 1-MeOH viewed perpendicular to the (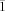
01) plane with (01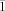) plane shown in red. Colours are as in Scheme 2. Hydrogen atoms not shown.

The crystal of **1-A^HT^** was then exposed to MeOH vapour in a sealed container maintained at 251 K. After 24 h, unit cell determination confirmed uptake of MeOH and conversion back to **1-MeOH**, in accord with previous observations.[16b] Optical images demonstrate that the small crack became larger during this process. This crack lies parallel to the (01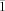) planes in **1-MeOH**, which corresponds to the plane that contains the same fluorous regions as noted previously (Figure 8; **1-MeOH** is indexed differently to **1-A^HT^**).

**Figure 9:**
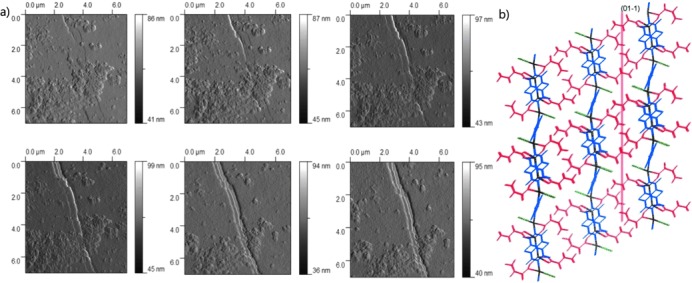
a) Sequence of AFM images of a 7 μm square of the (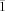
01) face of a single crystal of 1-MeOH recorded at 308 K in tapping mode at 8 min intervals during the release of MeOH. b) View of the crystal structure of 1-MeOH perpendicular to the (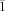
01) face (as for the AFM images) with (01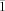) plane indicated as a red line. Colours as in Scheme 2. Hydrogen atoms not shown.

In a separate experiment a series of AFM images of a single crystal of **1-MeOH** maintained at a temperature of 308 K were recorded by scanning the (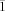
01) face. Over a period of 48 min a large crack developed in a direction corresponding to the (01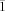) planes (Figure 9) as MeOH is lost. This observation is consistent with the optical microscope images.

## Discussion

We have previously shown that the crystalline coordination polymers [(Ag_4_(O_2_C(CF_2_)_2_CF_3_)_4_(TMP)_3_(ROH)_2_] (**1-ROH**; R=Me, Et, *i*Pr) liberate the coordinated alcohol (ROH) upon mild heating and are converted to the structurally-related coordination polymer [(Ag_4_(O_2_C(CF_2_)_2_CF_3_)_4_(TMP)_3_] (**1**) in a single-crystal-to-single-crystal manner, a process that involves cleavage and formation of covalent bonds as well as cleavage of hydrogen bonds. The process is reversible multiple times within one single crystal.[16b] Gas-phase IR spectroscopic measurements now establish that crystalline **1-ROH** exists in a solid–vapour equilibrium with the corresponding alcohol vapour (and crystalline **1**) when in a closed container. Equilibrium constants are similar for the three different systems, the value for **1-EtOH** being a factor of five smaller than those for **1-MeOH** and **1-*i*PrOH**. It is also noted from TGA-DSC data that loss of EtOH is completed at a higher temperature (380 K) than loss of MeOH or *i*PrOH (350 K), albeit with a slightly smaller measured enthalpy. There is no apparent correlation of the thermodynamic data with packing coefficients of the three compounds **1-ROH**, which all lie in the range 67.1–68.8 % (cf. 67.1 % for **1-A^HT^**).[21] The 1D coordination polymer **1** can be further converted irreversibly into the 2D coordination polymer [(Ag_4_(O_2_C(CF_2_)_2_CF_3_)_4_(TMP)_2_] (**2**) as a crystalline powder through selective loss of all singly-bridging TMP ligands (Scheme 2). Prior to the present study, however, our understanding of the mechanism(s) of this sequence of solid–vapour reactions was limited.

The earlier investigations in our laboratory focussed on a series of reactions involving one single crystal, in the sequence **1-EtOH**→**1**→**1-MeOH**→**1**→**1-*i*PrOH**→**1→1-EtOH**, in which, although alcohol removal reactions were conducted at elevated temperatures (320 K) and alcohol uptake by the crystal was conducted at reduced temperatures (248 K), crystal structure determinations at all stages in the reaction sequence were determined at a single temperature, 240 K. The crystal structure determined for coordination polymer **1** was identical at each stage of the reaction. The current investigation has revealed that coordination polymer **1** can actually exist as four polymorphs in the temperature range 100–340 K, comprising two polymorph types (**A** and **B**), each with a high-temperature and a low-temperature form, identified as **1-A^LT^**, **1-A^HT^**, **1-B^LT^** and **1-B^HT^**. Polymorph types **A** and **B** differ in the arrangement of the polymer tapes, whereas the HT and LT forms differ in the conformation of the fluoroalkyl chains and in their symmetry (*Z* ′ value).

The previously determined single crystal structure of **1**, denoted here as polymorph **1-A^HT^**, was obtained in the present work upon heating a single crystal of **1-EtOH** to remove EtOH; subsequently cooling the crystal below 115 K converts this to a new polymorph, **1-A^LT^**. However, in contrast to our earlier study, heating a single crystal of **1-*i*PrOH** generated a new polymorph, **1-B^HT^** rather than **1-A^HT^**, although transition via **1-A^HT^** to **1-B^HT^** cannot be ruled out; cooling the crystal of **1-B^HT^** below 250 K results in conversion to the fourth polymorph, **1-B^LT^**. Crystal structures of all polymorphs exhibit some disorder in the fluoroalkyl chains and individual chains have been identified in linear (*anti*) or bent (*gauche*) conformations (see above). These observations suggest significant mobility of the fluoroalkyl chains, consistent with the hypothesis that motion of the fluoroalkyl chains provides a mechanism for transport of alcohol molecules within the crystals. In accord with this assertion, optical microscopy and AFM studies on single crystals of **1-MeOH**/**1-A^HT^** have identified that during the MeOH release (and uptake) processes cracks form parallel to the planes defined layers of interdigitated perfluoroalkyl chains.

In light of the crystallographic characterisation of the polymorphic forms of **1**, in situ powder diffraction studies of the release of EtOH from **1-EtOH** and of *i*PrOH from **1-*i*PrOH** can be considered along with our prior study of MeOH release from **1-MeOH**[16b] to provide further information on the mechanism of the overall reaction manifold **1-ROH**→**1**→**2** (Scheme 2; note that **1**→**2** is a polycrystalline rather than single-crystal transformation). The studies of **1-MeOH** and **1-EtOH** reveal the transformation to **2** involves the generation of both **1-A^HT^** and **1-B^HT^** polymorphs of **1** upon loss of the alcohol, but prior to loss of TMP. In the study of **1-*i*PrOH** only polymorph **1-B^HT^** was identified, but temporary presence of **1-A^HT^** cannot be ruled out. The variation in proportion of **1-A^HT^** and **1-B^HT^** during the course of the reaction initiated from **1-EtOH** (Figure 4 and Figures S7–S14 in the Supporting Information) suggests that interconversion between the two polymorphs occurs at the elevated temperatures, but this has not been independently confirmed. The overall trend from the studies of the three **1-ROH** compounds suggests gradual conversion of **1-A^HT^** to **1-B^HT^**, but cannot unambiguously establish whether loss of TMP ligands to form coordination polymer **2** occurs only from **1-B^HT^** or from either of the two HT polymorphs.

**Scheme 3:**
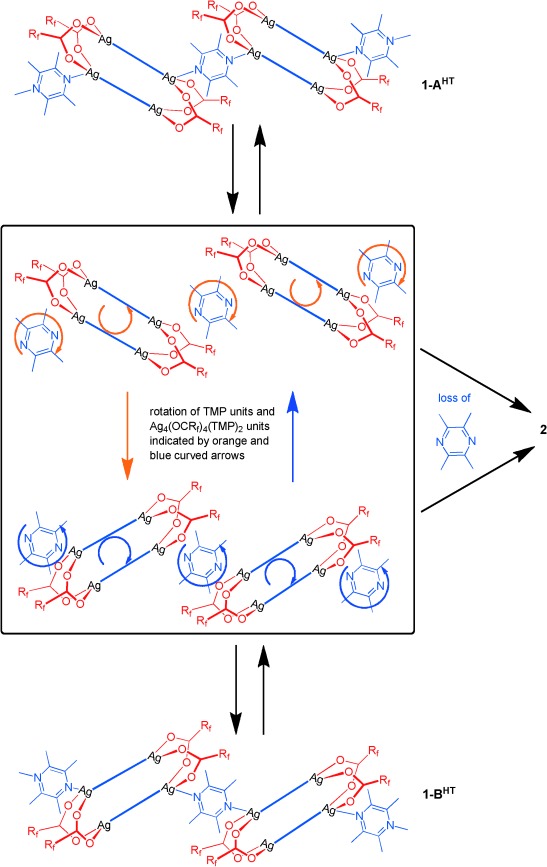
Proposed mechanism for conversion between polymorphs 1-A^HT^ and 1-B^HT^ involving dissociation of singly-bridging TMP ligands which leads to conversion of 1→2 by the loss of these dissociated TMP ligands. Conversion between polymorphs 1-A^HT^ and 1-B^HT^ requires no loss of dissociated TMP ligands, but instead requires rotation of these ligands and the residual Ag_4_(O_2_CR_f_)_4_(TMP)_2_ units (*R*_f_=CF_2_CF_2_CF_3_) in half of polymers in each crystal. The rotations are indicated by orange and blue curved arrows. The box indicates that the species depicted with dissociated TMP units correspond to a non-isolable intermediate or transition state species in the transformation of 1→2.

**Scheme 4:**
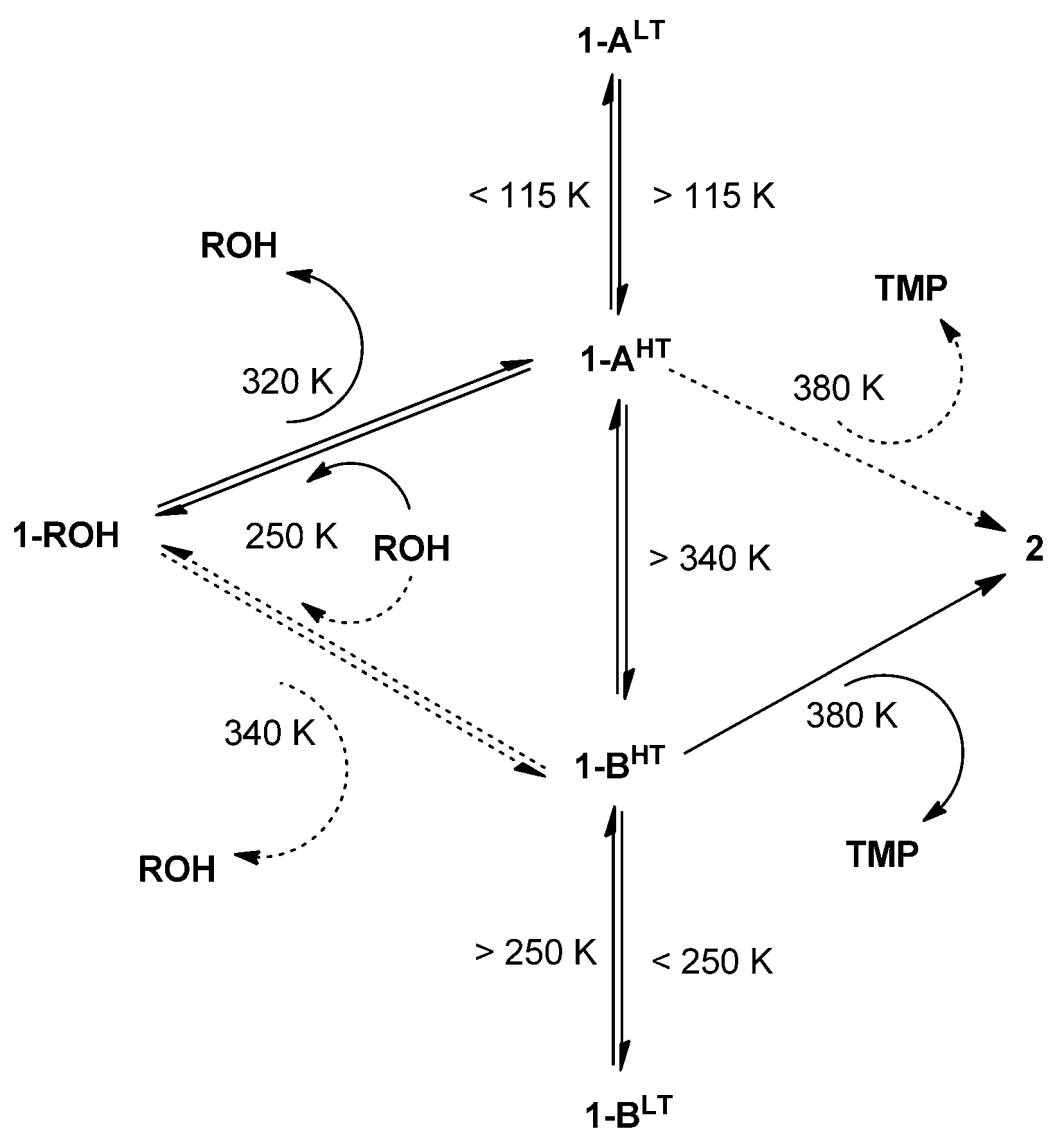
The proposed reaction manifold showing the conversion 1-ROH→[1-A^HT^⇌1-B^HT^]→2, the first two steps of which are equilibria in a closed environment. There is no definitive experimental evidence for the steps shown with dashed arrows, but the presence of these routes could not be ruled out. The conversions between the four polymorphs of 1, involving changes in temperature, are also indicated.

Conversion between polymorphs **1-A^HT^** and **1-B^HT^** involves reorienting half of the zigzag coordination polymers within the crystal so as to change the relative orientation of the Ag_4_(O_2_CR_f_)_4_(TMP)_2_ units (*R*_f_=CF_2_CF_2_CF_3_) and the connecting TMP units (Figure 3). This could be envisaged as requiring a 180° rotation of every other polymer about its principal axis. This requires a large motion, particularly involving the fluoroalkyl groups, which would need to be displaced from one fluoroalkyl layer and then inserted into a neighbouring layer. An alternative and more probable process would involve cleavage of Ag–N bonds between the Ag_4_(O_2_CR_f_)_4_(TMP)_2_ units and the singly bridging TMP ligands, followed by a rotation of the separated Ag_4_(O_2_CR_f_)_4_(TMP)_2_ units in which fluoroalkyl groups move *within* their current fluoroalkyl layer, before reformation of Ag–N bonds in the new orientation (Scheme 3). It can be envisioned that the latter process leads to mobility of the dissociated TMP ligands and ultimately to the release of these ligands, permitting direct linking of Ag_4_(O_2_CR_f_)_4_(TMP)_2_ units through Ag–O bonds, resulting in formation of coordination polymer **2**. The most plausible overall mechanism is via the route **1-ROH**→[**1-A^HT^**⇌**1-B^HT^]**→**2** (Scheme 4), wherein the (inter)conversion between **1-A^HT^** and **1-B^HT^** leads to loss of TMP ligands over time.

## Conclusion

A combination of experiments comprising in situ X-ray diffraction, thermal analyses (TGA, DSC), gas-phase IR spectroscopy and microscopy (optical, AFM) has provided mechanistic insight into the chemically-rich reaction manifold of the crystalline coordination polymer [Ag_4_(O_2_C(CF_2_)_2_CF_3_)_4_(TMP)_3_] (**1**). Despite an absence of porosity, as conventionally defined, crystals of coordination polymer **1** can reversibly absorb and release small alcohols. This involves formation/breaking of coordination bonds and hydrogen bonds, and thereby interconversion with coordination polymers [Ag_4_(O_2_C(CF_2_)_2_CF_3_)_4_(TMP)_3_(ROH)_2_] (**1-ROH**) by means of a solid–vapour equilibrium, which has been quantified by gas-phase IR spectroscopy. Our assertion that alcohol transport occurs via the fluoroalkyl layers, which contain fluoroalkyl groups of variable conformation, is consistent with observations from both optical microscopy and AFM, which reveal the development of cracks in the crystals parallel to these layers upon alcohol uptake and release.

Coordination polymer **1** is shown to exist as four polymorphs related by changes in orientation of the polymer and changes in conformation of the perfluoroalkyl chains of the carboxylate ligands. The existence of these polymorphs can be attributed to the flexibility of the coordination polymer, which we have previously assigned as essential to the absorption and release of small molecules. The two high-temperature polymorphs **1-A^HT^** and **1-B^HT^** are formed under the conditions of the reaction that converts **1-ROH**→**1**→**2** as crystalline solids through sequential loss first of two equivalents of alcohol ROH, then one equivalent of the bridging ligand TMP selectively from the singly bridging sites. The proposed mechanism for conversion between **1-A^HT^** and **1-B^HT^** involves Ag–N bond cleavage/reformation and is implicated in release of the TMP ligands to enable (irreversible) conversion of 1D coordination polymer **1** into 2D coordination polymer **2**.

The behaviour observed in these non-porous coordination polymers involving a two-step reaction process (**1-ROH**→**1**→**2**) is distinct from two-step processes identified either in MOFs with permanent porosity[22] or in coordination polymers in which guest molecules are bound non-covalently[23] rather than by coordination bonds. The extensive nature of the reaction manifold observed, and its amenability to investigation by multiple experimental techniques, provides a highly valuable insight into the behaviour of such solid-state materials. The understanding provided by such studies is of relevance across a broader field that includes many designed materials based on coordination chemistry, most notably MOFs, and provides encouragement for future development of flexible and responsive materials, for example with applications in areas such as sensing and catalysis.

## Experimental Section

### General

All reagents were purchased from Aldrich or Alfa Aesar and used as received. IR spectra were measured using PerkinElmer FT-IR spectrum 1000 instrument. Solid-state spectra were obtained by using a universal ATR sampling accessory. Gas-phase spectra were measured by using a purposely designed sample cell as previously described.[14b] TGA and DSC data were obtained using a Mettler Toledo TGA-DSC STARe system. Elemental analyses were conducted by the Elemental Analysis Service in the Department of Chemistry at University of Sheffield.

### Synthesis

**[Ag_4_(O_2_C(CF_2_)_2_CF_3_)_4_(TMP)_3_(MeOH)_2_] (1-MeOH), [Ag_4_(O_2_C(CF_2_)_2_CF_3_)_4_(TMP)_3_(EtOH)_2_] (1-EtOH), [Ag_4_(O_2_C(CF_2_)_2_CF_3_)_4_(TMP)_3_(*i*PrOH)_2_] (1-*i*PrOH), [Ag_4_(O_2_C(CF_2_)_2_CF_3_)_4_(TMP)_3_] (1) and [Ag_4_(O_2_C(CF_2_)_2_CF_3_)_4_(TMP)_2_] (2)**: Syntheses were conducted as previously reported.[16b] All compounds are characterised by elemental analysis and IR spectroscopy and phase purity was analysed by X-ray powder diffraction. Details are provided in the Supporting Information.

**[Ag(CO_2_(CF_2_)_2_CF_3_)TMP] (3)**: Silver(I) heptafluorobutanoate (166 mg, 0.517 mmol) was dissolved in acetonitrile (2 mL) and carefully layered on a solution of TMP (75 mg, 0.550 mmol) in acetonitrile (2 mL). Diffusion between layers at room temperature afforded colourless block crystals in 67 % yield within 2 days. Elemental analysis calcd (%): C 31.52, H 2.62, N 6.12; found: C 31.99, H 2.96, N 6.69. The crystal structure of **3** was determined by single-crystal X-ray diffraction.

### Single-crystal X-ray diffraction

**Data collection**: Synchrotron X-ray data were collected at a temperature of 100 K for **1-A^LT^** and 150 K for **1-B^LT^**, both polymorphs of **1**, formed by single-crystal-to-single-crystal transformations involving loss of alcohol molecules from **1-EtOH** and **1-*i*PrOH**, respectively (vide infra). The unit cell was determined for polymorph **1-B^HT^**, also formed in the solid-state transformation involving loss of *i*PrOH from **1-*i*PrOH**. Data were measured at beamline 9.8 (*λ*=0.6710 Å) at SRS, STFC Daresbury Laboratory, using a Bruker APEX II diffractometer equipped with an Oxford Cryosystems Cobra Plus nitrogen flow gas system. X-ray data for compound **3**, crystals of which were obtained directly from solution-phase synthesis, were collected at 150 K using Mo_Kα_ radiation on a Bruker SMART APEX II diffractometer equipped with an Oxford Cryosystems Cobra nitrogen-flow gas system.

**Crystal structure determination and refinement**: X-ray data were corrected for absorption using empirical methods (SADABS) based upon symmetry-equivalent reflections combined with measurements at different azimuthal angles.[24] All crystal structures were solved and refined against all *F*^2^ values using the SHELXTL suite of programs.[25] Non-hydrogen atoms were refined anisotropically where possible, whereas hydrogen atoms were placed in calculated positions, refined using idealised geometries (riding model) and assigned fixed isotropic displacement parameters. In many of the structure determinations fluoroalkyl chains are described as disordered over two orientations with carbon and fluorine atoms modelled using isotropic displacement parameters. A summary of the data collection and structure refinement information for **1-A^LT^**, **1-A^HT^**,[16b] **1-B^LT^** and **3**, as well as unit cell parameters for **1-B^HT^**, is provided in Table 3. Crystal structures of compounds **1-MeOH**, **1-EtOH**, **1-*i*PrOH** and **2** were reported previously,[16b] and are discussed here in the context of the solid–vapour reactions. Crystal data for these compounds are compiled in Table S1 in the Supporting Information. The triclinic unit cell for **1-A^HT^** is indexed such that unit cell dimensions are similar to those of the compounds **1-ROH**. However, this requires that the orientation of the coordination polymers with respect to the unit cell axes is different for **1-A^HT^** than for **1-ROH** (vide infra). Crystal structures of **1-ROH**, **1-A^HT^** and **2** are shown in Scheme 2. Crystal structures of **1-A^LT^** and **1-B^LT^** are shown in Figure 2, along with the two high temperature polymorphs, **1-A^HT^** and **1-B^HT^**, for comparison. The crystal structure of **3** is shown in Figure S1 in the Supporting Information.

**Table 3:** Data collection, structure solution, and refinement parameters for 1-A^LT^, 1-A^HT^, 1-B^LT^ and 3. Unit cell parameters for 1-B^HT^

	1-A^LT^	1-A^HT[a]^	1-B^LT^	1-B^HT[b]^	3
crystal colour	colourless	colourless	colourless	colourless	colourless
crystal size [mm]	0.12×0.12×0.11	0.28×0.28×0.27	not recorded	not recorded	0.40×0.35×0.20
crystal system	triclinic	triclinic	monoclinic	monoclinic	monoclinic
space group, *Z*	*P* 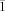 , 2	*P* 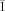 , 1	*P*2_1_/*c*, 4	*P*2_1_/*c*, 2	*P*2_1_/*c*, 4
*a* [*Å*]	12.8731(1)	8.5215(2)	28.258(6)	22.75(1)	13.1680(6)
*b* [*Å*]	14.8594(1)	12.9026(3)	8.4349(2)	8.573(3)	9.4003(4)
*c* [*Å*]	16.8199(1)	14.8408(4)	24.343(5)	15.102(7)	12.9359(6)
*α* [°]	107.2410(1)	112.854(1)	90	90	90
*β* [°]	107.2690(1)	90.486(1)	111.445(2)	100.48(8)	107.137(2)
*γ* [°]	105.8020(1)	109.325(1)	90	90	90
*V* [Å^3^]	2695.6(5)	1405.34(6)	5400(2)	2897(2)	1530.1(1)
*ρ* [Mg m^−3^]	2.085	2.000	2.081	1.940	1.984
*λ* [Å]	0.6710	0.71073	0.6710	0.6710	0.71073
*T* [K]	100	240	150	340	150
*μ*(Mo_Kα_) [mm^−1^]	1.365	1.519	1.362		1.404
*θ* range [°]	1.47–25.82	1.50–27.54	2.32–23.84		1.62–27.57
reflns collected	25 166	25 544	49 205		13 661
independent reflns (*R*_int_)	12 242 (0.0383)	6404 (0.0296)	9765 (0.1756)		3499 (0.0232)
reflns used in refinement, *n*	12 242	6404	9765		3499
L.S. parameters, *p*	824	376	779		221
no. of restraints, *r*	216	295	204		0
*R*1 (*F*)^[c]^ *I*>2.0σ*(I)*	0.0466	0.0730	0.0863		0.0261
*wR*2(*F*^2^),^[c]^ all data	0.1374	0.1945	0.2297		0.0754
*S*(*F*^*2*^),^[c]^ all data	1.065	1.085	1.028		0.951

[a] Crystal structure from reference [16b] (therein identified simply as compound **1**). [b] Only sufficient data were measured to determine unit cell dimensions and space group. Structure was determined from powder diffraction data (see below). [c] *R*1(*F*)=Σ(|*F_o_*|−|*F_c_*|)/Σ|*F_o_*|; *wR*^2^(*F*^2^)=[Σ*w*(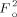
−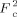)^2^/Σ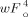]^1/2^; *S*(*F*^2^)=[Σ*w*(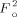
−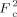)^2^/(*n*+*r*−*p*)]^1/2^.

CCDC 1044595 http://www.ccdc.cam.ac.uk/cgi-bin/catreq.cgi, 1044596 http://www.ccdc.cam.ac.uk/cgi-bin/catreq.cgi, 1044597 http://www.ccdc.cam.ac.uk/cgi-bin/catreq.cgi, 1044598 http://www.ccdc.cam.ac.uk/cgi-bin/catreq.cgifor compounds **1-A^LT^**, **1-B^LT^**, **1-B^HT^** and **3** contain the supplementary crystallographic data for this paper. These data can be obtained free of charge from The Cambridge Crystallographic Data Centre via http://www.ccdc.cam.ac.uk/data_request/cif.

### Single-Crystal-to-Single-Crystal Transformations (SCSCT)

**SCSCT of 1-MeOH→1-A^HT^→1-MeOH**: X-ray data for unit cell determination and a series of optical images were collected during the single-crystal-to-single-crystal reaction sequence **1-MeOH→1-A^HT^→1-MeOH**. A Bruker SMART APEX II diffractometer was used with Mo_Kα_ radiation and an Oxford Cryosystems Cobra nitrogen flow gas system used for heating and cooling the crystal. A crystal of the starting compound **1-MeOH** was affixed to a glass fibre with a minimum of adhesive on one side of the crystal. A partial data set was collected at room temperature confirming the structure by unit cell determination. The unit cell was monitored at room temperature for 210 min, after which the crystal could be indexed as a single phase of compound **1-A^HT^**, resulting from complete loss of MeOH. A series of optical images of the crystal were recorded using a CCD microscope with viewing directions established from the orientation matrix. The crystal was then exposed to methanol vapours for 24 hrs at 251 K in a sealed container, which resulted in full conversion to compound **1-MeOH** by uptake of MeOH. A partial data set was then obtained confirming conversion to **1-MeOH** by unit cell determination and a further series of optical images were recorded.

**SCSCT of 1-EtOH→1-A^HT^→1-A^LT^→1-A^HT^→1-A^LT^**: An in situ experiment was undertaken to investigate the removal of EtOH from a single crystal of **1-EtOH** at beamline 9.8 (*λ*=0.6710 Å), SRS, STFC Daresbury Laboratory, using a Bruker APEX II diffractometer equipped with an Oxford Cryosystems Cobra nitrogen flow gas system. A single crystal of **1-EtOH** was heated to 318 K for 24 h, after which, consistent with previous experiments,[16b] it had transformed into coordination polymer **1** as a single crystal in the polymorphic form **1-A^HT^**. The crystal was cooled to 100 K, after which repeated determinations of the unit cell dimensions confirmed that it had transformed into a new form, hereafter referred to as **1-A^LT^**. A full data set was obtained and a crystal structure determination of new polymorph **1-A^LT^** was undertaken. The crystal was subsequently heated to room temperature, then cooled, first to 125 K and subsequently to 115 K, during which a unit cell determination was undertaken at each stage. The crystal had reverted to polymorph **1-A^HT^** and remained so within this temperature range (i.e. 115<T<295 K). Finally, the crystal was cooled to 110 K, whereupon determination of the unit cell dimensions established that the crystal was once again polymorph **1-A^LT^**, thereby confirming the reversibility of the phase transition.

**SCSCT of 1-*i*PrOH→1-B^HT^→1-B^LT^**: An in situ experiment was undertaken to investigate the removal of *i*PrOH from a single crystal of **1-*i*PrOH** at beamline 9.8 (*λ*=0.6710 Å), SRS, STFC Daresbury Laboratory, using a Bruker APEX II diffractometer equipped with an Oxford Cryosystems Cobra nitrogen flow gas system. An initial unit cell determination confirmed the starting crystal as **1-*i*PrOH**. The crystal was heated at 320 K for 60 min and then at 340 K for 40 min, after which repeated determinations of the unit cell dimensions indicated that the crystal had transformed into a new form, later determined to be a new polymorph of **1**, hereafter referred to as polymorph **1-B^HT^**. The crystal was cooled to 250 K, at which stage a unit cell determination indicated that the crystal remained as polymorph **1-B^HT^**. Finally, the crystal was cooled to 150 K, after which repeated determinations of the unit cell dimensions confirmed transformation into polymorph **1-B^LT^**. A complete data set was obtained and a crystal structure determination of polymorph **1-B^LT^** was undertaken.

### Powder X-ray diffraction (PXRD)

**Loss of MeOH from 1-MeOH**: In situ PXRD data for this reaction have been previously reported[16b] and are summarised in Figure S6 in the Supporting Information.

**Loss of alcohol from 1-EtOH and 1-*i*PrOH**: The ethanol and isopropanol release from compounds **1-EtOH** and **1-*i*PrOH**, respectively, was monitored in situ using synchrotron X-ray powder diffraction (Figures 4 and 5). The white microcrystalline compounds **1-EtOH** and **1-*i*PrOH** were each loaded into a 0.7 mm borosilicate capillary and X-ray diffraction data were collected (*λ*=0.826741(1) Å) at beamline I11 at Diamond Light Source,[26] equipped with a wide-angle (90°) PSD detector comprising 18 Mythen-2 modules.[26b] The temperature was increased using an Oxford Cryosystems Cryostream Plus from 295 K to 340 K for compound **1-EtOH** and to 373 K for compound **1-*i*PrOH**. A series of patterns were collected with 5 s exposures at intervals of 20 min. Rietveld refinement[18] was performed for each pattern using the TOPAS program,[27] revealing a fit to as few as one or as many as four phases (from **1-EtOH**, **1-A^HT^**, **1-B^HT^**, **2** and **3** or **1-*i*PrOH**, **1-B^HT^** and **2**) that are present at different stages of the two reaction sequences (Figures S7–S14 and Figures S15–S17 in the Supporting Information). The impurity phase **3** was found in the material synthesised as **1-EtOH**, but remains unchanged during the conversion of **1-EtOH** to **1** by loss of EtOH. The crystal structure of **3** was established by single-crystal X-ray diffraction following independent synthesis of this compound.

**Figure 10:**
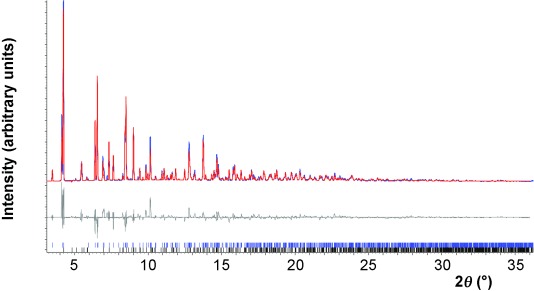
Observed (blue) and calculated (red) profiles and difference plot [(*I*_obs_−*I*_calcd_)] of the Rietveld refinement for X-ray powder diffraction pattern (2≤2*θ*≤36°, max. resolution 1.33 Å) at 295 K (*R_wp_*=0.14769, *R_wp_′*=0.30028 gof=17.132). Composition: 1-B^HT^ (80(1) %) and 2 (20(1) %). Upper tick marks refer to peaks for 1-B^HT^ and lower tick marks refer to peaks for 2.

**Structure determination of 1-B^HT^**: Upon full conversion of **1-*i*PrOH**, through loss of *i*PrOH, the final pattern measured after cooling to 295 K was found to contain predominantly **1-B^HT^** with small amount of **2**, for which the crystal structure is known.[16b] The pattern was indexed and a two-phase Pawley fit was conducted, resulting in unit cell parameters for **1-B^HT^** that compare well to those determined by single-crystal diffraction (Table 3). Structure determination of polymorph **1-B^HT^** was then successfully accomplished by direct space methods using the TOPAS-academic program. The unit Ag_2_(CO_2_(CF_2_)_2_CF_3_)_2_(TMP) and half a molecule of TMP were introduced as rigid bodies. Each of these two groups is situated about a crystallographic inversion centre. A dummy atom was added to each rigid body and placed at the inversion centres of the unit cell. The rigid bodies were refined with rotational freedom. A common thermal parameter was refined for all the atoms. Spherical-harmonic correction of the intensities for preferred orientation was applied in the final stage of refinement. A two-phase Rietveld refinement converged to *R*_wp_=0.14769, 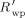
=0.30028 (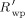 is the background subtracted *R*_wp_) (Figure 10).

### Thermogravimetric analysis (TGA) and differential scanning calorimetry (DSC)

Samples of **1-MeOH**, **1-EtOH** and **1-*i*PrOH** were heated at 2 °C min^−1^ over the temperature range 25–400 °C under a flow of dry N_2_ gas and monitored simultaneously by TGA and DSC measurements. Enthalpies for the loss of alcohol and the loss of TMP were calculated by integration of the endothermic peaks in the DSC traces.

### Atomic force microscopy—monitoring release of methanol by crystals of 1-MeOH

AFM measurements were conducted using an Asylum MFP-3D-SA instrument (Asylum Research, Santa Barbara, CA, USA). Crystals of compound **1-MeOH** were transferred to a specimen disk (Figure S18 in the Supporting Information) that had been coated with partially cured (approx. 45 s under a Blak-RayB100 bulb at a distance of 15 cm) UV-curable thiolene adhesive (NOA-81, Norland Products, Inc.). The optical cement was completely cured by exposing the specimen to UV radiation for another 2 min. The AFM experiments were run in tapping mode because of the softness of the surface of the crystal. The azimuthal orientation of the crystals with respect to the AFM image frame was identified using an optical microscope above the AFM cell. In situ AFM was performed in a PolyHeater heating stage (Asylum Research, Santa Barbara, CA, USA) equipped with a customised anodised aluminium insert using a Si_3_N_4_ cantilever tip with an aluminium reflector coating and a force constant of approximately 2 N m^−1^.

### Gas-phase Fourier-transform infrared spectroscopy (FTIR)

Fourier transform infrared (FTIR) spectroscopic experiments were conducted at 22 °C using a double-walled 10 cm glass IR absorption cell fitted with either KCl or KBr windows and a suspended sample container (Figure S19 in the Supporting Information) as previously described.[14b] To obtain time-dependent concentrations of methanol, ethanol and isopropanol vapours, gas-phase IR spectra in the region of 400–4000 cm^−1^ were acquired using a FTIR spectrometer (PerkinElmer Paragon 1000, resolution 1 cm^−1^, no apodisation). The spectrometer was operated in the single-beam mode, that is, sample and background (empty cell) spectra were recorded separately. The area under the methanol C-O stretching absorption band was integrated from 950 to 1100 cm^−1^ after background subtraction and baseline correction to determine the partial pressure of methanol in accordance with prior calibration (Figures S20 and S21 in the Supporting Information). For ethanol and isopropanol, the areas under the C–O stretching and C–H bending absorption bands, which overlap, were integrated from 950 to 1175 cm^−1^ and 900 to 1000 cm^−1^, respectively, after background subtraction and baseline correction, to determine the partial pressures of ethanol and isopropanol in accordance with prior calibrations[14b] (Figures S22–S25 in the Supporting Information).

The absorption cell was loaded with a 50 mg sample of polycrystalline compound **1-MeOH**, **1-EtOH** or **1-*i*PrOH**, and in a separate experiment with 100 mg **1-MeOH**. The cell was connected to a vacuum line to remove all the H_2_O and other gases present. Sixteen scans were accumulated over a period of 2 min for each IR spectrum to provide a satisfactory signal-to-noise ratio. Additional scans to improve accuracy further were not made since continuous evolution of methanol, ethanol or isopropanol vapour was expected. Measurements were continued until no increase in intensity was observed for the absorption bands monitored.

## Supporting Information

As a service to our authors and readers, this journal provides supporting information supplied by the authors. Such materials are peer reviewed and may be re-organized for online delivery, but are not copy-edited or typeset. Technical support issues arising from supporting information (other than missing files) should be addressed to the authors.

miscellaneous_information
