# Supplementary material for: Coordination Polymer Flexibility Leads to Polymorphism and Enables a Crystalline Solid–Vapour Reaction: A Multi-technique Mechanistic Study
Source: Chemistry. 2015 May 11;21(24):8799–811. doi: 10.1002/chem.201500514 (PMC4641464; doi:10.1002/chem.201500514)
Supplement: Supplementary file 1 — miscellaneous_information [file chem0021-8799-sd1.pdf]

CHEMISTRY
A European Journal

Supporting Information

Coordination Polymer Flexibility Leads to Polymorphism and Enables a Crystalline Solid–Vapour Reaction: A Multi-technique Mechanistic Study

Iñigo J. Vitórica-Yrezábal,[a, g] Stefano Libri,[a] Jason R. Loader,[a]
Guillermo Mínguez Espallargas,[b] Michael Hippler,[a] Ashleigh J. Fletcher,[c]
Stephen P. Thompson,[d] John E. Warren,[e] Daniele Musumeci,[f, h] Michael D. Ward,[f] and
Lee Brammer*[a]

chem_201500514_sm_miscellaneous_information.pdf

Supplementary material

Coordination polymer flexibility leads to polymorphism and permits crystalline solid-vapour reaction: a multi-technique mechanistic study†

Iñigo J. Vitórica-Yrezábal,[1,§] Stefano Libri,[1] Jason R. Loader,[1] Guillermo Mínguez Espallargas,[2] Michael Hippler,[1] Ashleigh J. Fletcher,[3] Stephen P. Thompson,[4] John E. Warren,[5] Daniele Musumeci,[6,‡] Michael D. Ward[6] and Lee Brammer*,[1]

Email: lee.brammer@sheffield.ac.uk

[1]*Department of Chemistry, University of Sheffield, Sheffield S3 7HF, UK.*
[2]*Instituto de Ciencia Molecular (ICMol), Universidad de Valencia, c/ Catedrático José Beltrán, 2, 46980 Paterna, Spain.*
[3]*Department of Chemical and Process Engineering, University of Strathclyde, 75 Montrose St, Glasgow G1 1XJ, Scotland.*
[4]*Diamond Light Source, Harwell Science and Innovation Campus, Didcot, Oxfordshire OX11 0DE, UK*
[5]*School of Materials, University of Manchester, Manchester M13 9PL, UK.*
[6] *Molecular Design Institute, NYU Department of Chemistry, 100 Washington Square East, New York, NY 10003, USA.*

§ *Current address: School of Chemistry, University of Manchester, Oxford Road, Manchester, M13 9PL, UK.*
‡ *Current address: Department of Chemistry, York College of The City University of New York, 94-20 Guy R. Brewer Blvd, Jamaica, New York, NY 11451, USA.*

Table of Contents

Syntheses of **1-MeOH**, **1-EtOH**, **1-*i*PrOH**, **2** and **3** — Page S2
Single crystal diffraction data for **1-MeOH**, **1-EtOH**, **1-^{i}PrOH** and **2** — Page S3
Crystal structure of **3** — Page S3
Crystal structures of polymorphs **1-A^{LT}**, **1-B^{LT}**, **1-A^{HT}** and **1-B^{HT}** — Page S4
Powder diffraction: phase purity of **1-MeOH**, **1-EtOH** and **1-^{i}PrOH** — Page S5
In situ powder diffraction:
1-MeOH → 1-A^{HT} + 1-B^{HT} → 1-A^{HT} + 1-B^{HT} + 2 → 1-B^{HT} + 2 → 2 — Page S6
1-EtOH → 1-A^{HT} + 1-B^{HT} (Rietveld fits) — Page S6
1-^{i}PrOH → 1-B^{HT} (Rietveld fits) — Page S10
AFM experiments: monitoring release of methanol by crystals of **1-MeOH** — Page S11
Gas-phase Fourier-transform Infra-red spectroscopy (FTIR) — Page S11
TGA and DSC traces: **1-MeOH, 1-EtOH, 1-*i*PrOH** — Page S17
References — Page S19

Synthesis of $[Ag_4(O_2C(CF_2)_2CF_3)_4(TMP)_3(MeOH)_2]_n$ (1-MeOH). Silver(I) heptafluorobutyrate (96 mg, 0.30 mmol) was dissolved in methanol (1.5 mL) and carefully layered on a solution of 2,3,5,6-tetramethylpyrazine (TMP) (30 mg, 0.220 mmol) in dichloromethane (DCM) solution (1 mL). Diffusion between layers at 5 °C afforded colourless block crystals in 81% yield within 3 days. Anal. Calc. ($C_{42}H_{44}F_{28}N_6O_{10}Ag_4$): C, 28.72; H, 2.52; N, 4.78%; found C, 28.59; H, 2.14; N, 4.91%. IR data (cm^{-1}) ν(OH) 3312, $\nu_{sym}(CO_2)$ 1412, $\nu_{asym}(CO_2)$ 1663. Phase purity was confirmed by Pawley refinement[S1] of unit cell parameters against X-ray powder diffraction data (see Figure S2).

Synthesis of $[Ag_4(O_2C(CF_2)_2CF_3)_4(TMP)_3(EtOH)_2]_n$ (1-EtOH). Silver(I) heptafluorobutyrate (96 mg, 0.30 mmol) was dissolved in ethanol (1.5 mL) and carefully layered on a solution of TMP (30 mg, 0.220 mmol) in DCM solution (1 mL). Diffusion between layers at 5 °C afforded colourless block crystals in 83% yield within 3 days. Anal. Calc. ($C_{44}H_{48}F_{28}N_6O_{10}Ag_4$): C, 29.62; H, 2.71; N, 4.71%; found C, 29.53; H, 2.32; N, 4.40%. IR data (cm^{-1}) ν(OH) 3323, $\nu_{sym}(CO_2)$ 1411, $\nu_{asym}(CO_2)$ 1663. Phase purity was confirmed by Rietveld refinement[S1] against X-ray powder diffraction data (see Figure S3).

Syntheses of $[Ag_4(O_2C(CF_2)_2CF_3)_4(TMP)_3(\textit{i}PrOH)_2]_n$ (1-*i*PrOH). Silver(I) heptafluorobutyrate (96 mg, 0.30 mmol) was dissolved in isopropanol (1.5 mL) and carefully layered on a solution of TMP (30 mg, 0.220 mmol) in DCM solution (1 mL). Diffusion between layers at 5 °C afforded colourless block crystals in 67% yield within 3 days. Anal. Calc. ($C_{46}H_{52}F_{28}N_6O_{10}Ag_4$): C, 30.48; H, 2.89; N, 4.64%; found C, 29.56; H, 2.48; N, 4.53%. Repeated syntheses indicate that $[Ag_4(O_2C(CF_2)_2CF_3)_4(TMP)_2]_n$ (**2**) can be formed as a by-product. The disagreement of the elemental analysis is likely due to the presence of a small amount of **2**. IR data (cm^{-1}) ν(OH) 3357, $\nu_{sym}(CO_2)$ 1413, $\nu_{asym}(CO_2)$ 1663. Alternatively, **(1-*i*PrOH)** can be prepared in quantitative yield more reliably by exposure of **1** (polymorph **1-A^{HT}**) to *i*PrOH vapour. Thus, $[Ag_4(O_2C(CF_2)_2CF_3)_4(TMP)_3]$ **1-A^{HT}** (100 mg, 0.0612mmol) was exposed to *i*PrOH at –20°C for 3 weeks, after which time colourless needles of **1-*i*PrOH** were obtained in quantitative yield (as shown by Rietveld fit to the X-ray powder diffraction pattern – Figure S4). Calc. ($C_{46}H_{52}F_{28}N_6O_{10}Ag_4$): C, 30.48; H, 2.89; N, 4.64%. Found: C, 30.00; H, 2.95; N, 4.39%.

Syntheses of $[Ag_4(O_2C(CF_2)_2CF_3)_4(TMP)_2]_n$ (2). Silver(I) heptafluorobutyrate (166 mg, 0.517 mmol) was dissolved in n-butanol (2 mL) and carefully layered on a solution of TMP (60 mg, 0.440 mmol) in DCM solution (2 mL). Diffusion between layers at 5 °C afforded colourless plate crystals in 34% yield within 2 days. Calc.: C, 24.69; H, 1.54; N, 3.60%. Found: C, 24.62; H, 1.28; N, 3.47%. IR data (cm^{-1}) $\nu_{sym}(CO_2)$ 1401, $\nu_{asym}(CO_2)$ 1648. The product was characterized by single crystal X-ray diffraction. Alternatively, **2** was synthesized by heating **1-EtOH** to remove EtOH and TMP. **1-EtOH** (100 mg, 0.0499 mmol) was placed in a flask a heated at 80° C for three hours. White crystals of **2** formed quantitatively. Calc. ($C_{32}H_{24}F_{28}N_4O_8Ag_4$): C, 24.69; H, 1.54; N, 3.60%. Found: C, 24.94; H, 1.60; N, 3.35%. IR data (cm^{-1}) $\nu_{sym}(CO_2)$ 1401, $\nu_{asym}(CO_2)$ 1649.

$[Ag(CO_2(CF_2)_2CF_3)TMP]$ (3). Silver(I) heptafluorobutanoate (166 mg, 0.517 mmol) was dissolved in acetonitrile (2 mL) and carefully layered on a solution of TMP (75 mg, 0.550 mmol) in acetonitrile solution (2 mL). Diffusion between layers at room temperature afforded colourless block crystals in 67% yield within 2 days. Calc.: C, 31.52; H, 2.62; N, 6.12%. Found: C, 31.99; H, 2.96; N, 6.69%. The crystal structure of **3** was determined by single crystal X-ray diffraction

Single Crystal X-ray Diffraction

Table S1. Data Collection, Structure Solution, and Refinement Parameters for **1-MeOH**, **1-EtOH**, **1-*i*PrOH** and **2** (data are reproduced from ref S2 for comparison with crystal structures reported in this paper)

	**1-MeOH**	**1-EtOH**	**1-*i*PrOH**	**2**
Crystal colour	colourless	colourless	colourless	colourless
Crystal size (mm)	0.22 x 0.18 x 0.08	0.28 x 0.28 x 0.27	0.27 x 0.18 x 0.12	0.28 x 0.22 x 0.04
Crystal system	triclinic	triclinic	triclinic	triclinic
Space group, *Z*	*P*-1, 1	*P*-1, 1	*P*-1, 1	*P*-1, 1
a (Å)	8.800(2)	8.8667(2)	8.9624(6)	10.614(4)
b (Å)	12.268(3)	12.0846(3)	12.0249(9)	11.051(4)
c (Å)	14.904(4)	15.4595(3)	15.360(2)	11.967(5)
α (°)	109.781(4)	109.451(1)	108.091(5)	71.73(2)
β (°)	96.709(4)	98.228(1)	96.976(5)	71.83(2)
γ (°)	104.522(4)	103.559(1)	103.206(4)	62.31(2)
V (Å^3)	1429.4(6)	1473.55(6)	1498.5(2)	1157.0(7)
Density (Mg.m^{-3})	2.040	2.011	2.008	2.233
Wavelength (Å)	0.71073	0.71073	0.71073	0.71073
Temperature (K)	150	150	150	150
μ(Mo-K$_\alpha$) (mm^{-1})	1.500	1.456	1.434	1.833
Θ range (°)	1.49 to 27.56	1.87 to 27.51	1.43 to 37.13	2.21 to 27.64
Reflns collected	16491	27606	51533	18039
Independent reflns (R_{int})	6418 (0.1325)	6757 (0.0222)	13972 (0.0331)	5261 (0.0854)
Reflns used in refinement, *n*	6418	6757	13972	5261
L.S. parameters, *p*	401	405	432	319
No. of restraints, *r*	0	62	0	66
$R1$ (F)[a] $I > 2.0\sigma(I)$	0.0632	0.0546	0.0422	0.0647
$wR2(F^2)$[a], all data	0.1670	0.1548	0.1177	0.1704
$S(F^2)$[a], all data	0.887	1.020	1.006	0.988

[a] $R1(F) = \Sigma(|F_o| - |F_c|)/\Sigma|F_o|$; $wR^2(F^2) = [\Sigma w(F_o{}^2 - F_c{}^2)^2/\Sigma wF_o{}^4]^{½}$; $S(F^2) = [\Sigma w(F_o{}^2 - F_c{}^2)^2/(n + r - p)]^{½}$

Crystal Structure of 3

Compound **3** has been synthesized as a colourless crystalline solid by diffusion between two acetonitrile solutions of silver(I) heptafluorobutanoate and TMP, respectively (see Experimental section). The coordination polymer forms a 1D zig-zag tape, in which silver atoms have a distorted tetrahedral geometry. Each Ag(I) centre is coordinated to one chelating heptafluorobutanoate and two TMP ligands (Figure S1). Thus, single TMP ligands link the $Ag(O_2C(CF_2)_2CF_3)$ units, forming a coordination polymer which extends in the [001] direction. The 1D zig-zag geometry of the

coordination polymer arises from the distorted tetrahedral coordination geometry of the Ag(I) ion, with an N–Ag–N angle of 133.48(7) °. The perfluoroalkyl chains are interdigitated in the (100) plane.

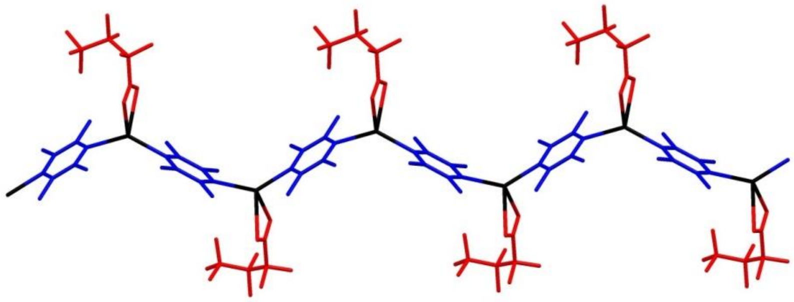

Figure S1. Crystal structure of $[Ag(O_2C(CF_2)_2CF_3)(TMP)]_n$ (**3**). Hydrogen atoms are not shown. Silver(I) centres in black; TMP ligands in blue; heptafluorobutanoate ligands in red.

Crystal structures of polymorphs 1-A^{LT}, 1-B^{LT}, 1-A^{HT} and 1-B^{HT}

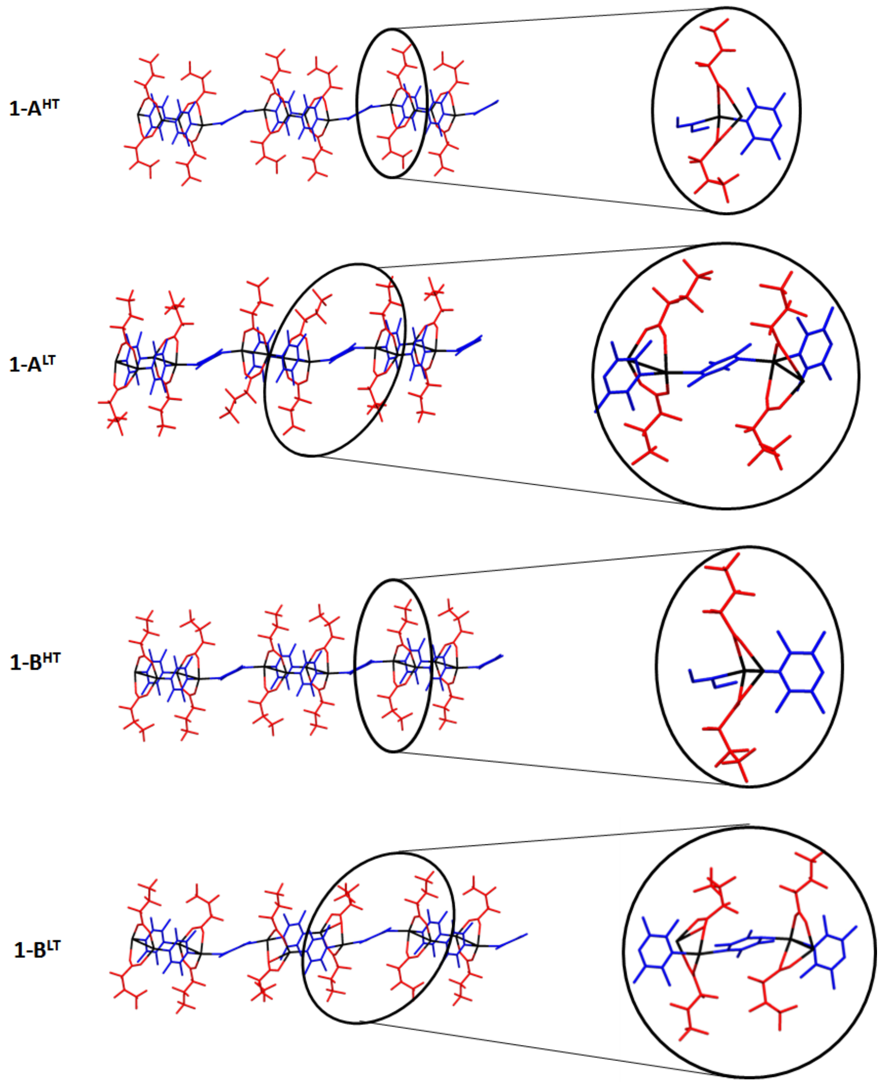

Figure S2. Crystal structures of the polymorphs (**1-A^{HT}**, **1-A^{LT}**, **1-B^{HT}** and **1-B^{LT}**) of coordination polymer **1** $[Ag_4(O_2C(CF_2)_2CF_3)_4(TMP)_3]_n$. Expansions provided corresponds to the crystallographic asymmetric units, showing: a) the coordination environment of the Ag(I) centres; b) the different conformational (*gauche-anti)* distribution of the perfluorocarboxylate chains. Hydrogen atoms are not shown in expanded views, and TMP ligands are only shown in part for polymorphs **1-A^{HT}** and **1-B^{HT}**. Colour code as in Figure S1.

Powder X-ray Diffraction

Phase purity of 1-MeOH. A polycrystalline sample of **1-MeOH** was lightly ground in an agate mortar and pestle and loaded into a 0.7 mm borosilicate glass capillary prior to being mounted and aligned on a Bruker-AXS D8 Advance powder diffractometer operating with Ge-monochromated Cu-$K_{\alpha 1}$ radiation (λ = 1.54056 Å). Powder patterns were measured at a scan rate no faster than 1 °/min in the range $4 \leq 2\theta \leq 40$ °. The powder pattern was indexed using the program TOPAS.[S3] A unit cell was found corresponding to crystal structure of 1-MeOH already established from single crystal diffraction. A Pawley refinement,[S4] conducted using TOPAS, was implemented, confirming the phase purity of **1-MeOH**. Pawley refinement converged to R_{wp} of 0.0518 , R_{wp}' = 0.1334 (R_{wp}' is the background subtracted R_{wp}); see Figure S3.

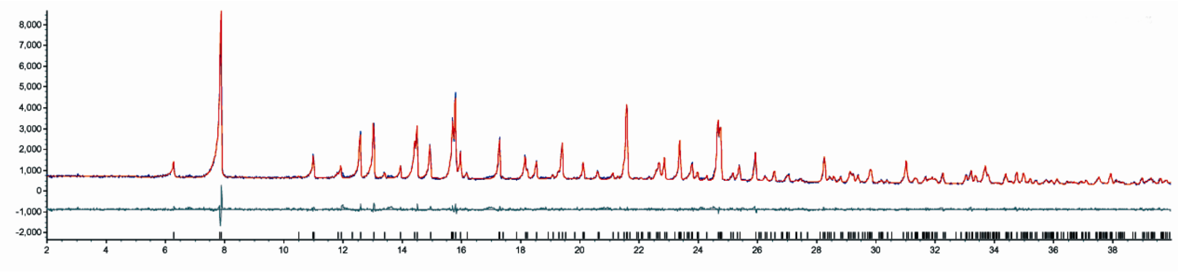

Figure S3. Observed (blue) and calculated (red) profiles and difference plot [I_{obs}-I_{calc}] (grey) of the Pawley refinement of **1-MeOH** (2θ range: 2.0 – 40.0°; maximum resolution 2.25 Å).

Phase purity of 1-EtOH. White microcrystalline **1-EtOH**, product of the solution phase synthesis, was loaded into a 0.7 mm borosilicate capillary and X-ray diffraction data were collected (λ= 0.799993(8) Å) at station ID31[S5] at the European Synchrotron Radiation Source (ESRF) using a 9-channel multi-analyser crystal (MAC) detector. All data were collected at room temperature. The powder pattern was indexed using the program TOPAS. A unit cell was found corresponding to crystal structure of **1-EtOH** already established from single crystal diffraction. The starting model used for Rietveld refinement, conducted using TOPAS, was the single crystal structure of **1-EtOH.** The model for the structure was refined with one global isotropic thermal parameter. A 6th order spherical harmonic correction of the intensities for preferred orientation was applied in the final stage of refinement. Rietveld refinement converged to R_{wp} of 0.14630 , R_{wp}' = 0.23936 (R_{wp}' is the background subtracted R_{wp}); see Figure S4.

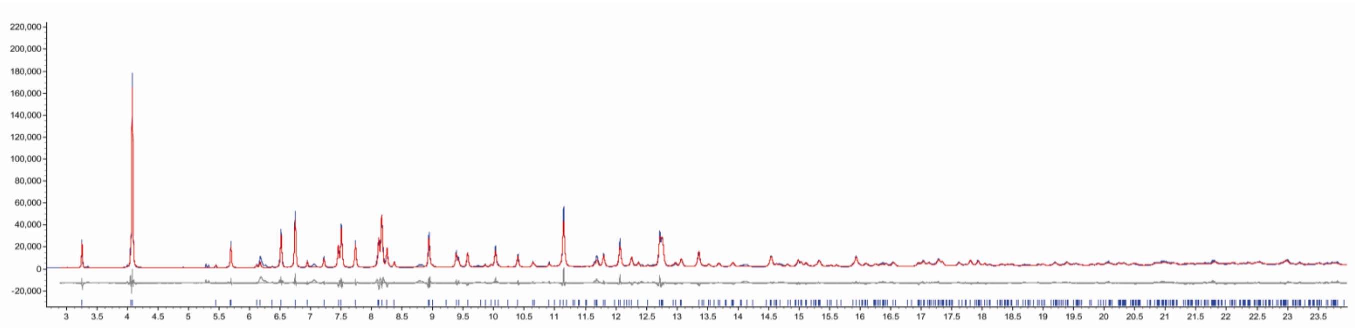

Figure S4. Observed (blue) and calculated (red) profiles and difference plot [I_{obs}-I_{calc}] (grey) of the Rietveld refinement of **1-EtOH**. (2θ range: 3.0 – 24°; maximum resolution 1.9238Å).

Phase purity of 1-*i*PrOH. White microcrystalline **1-*i*PrOH**, product of the solid-vapour synthesis from **1** and *i*PrOH, was loaded into a 0.7mm borosilicate capillary and X-ray diffraction data were collected (λ= 0.826741(1) Å) at beamline I11 at Diamond Light Source,[S6] equipped with a wide angle (90 °) PSD detector comprising 18 Mythen-2 modules.[S7] A series of 14 pairs of scans were

conducted at room temperature, each pair related by a 0.25 ° detector offset to account for gaps between detector modules. The resulting 28 patterns were summed to give the final pattern for structural analysis. The powder pattern was indexed using the program TOPAS. A unit cell was found corresponding to the known crystal structure of **1-*i*PrOH**. The starting model used for Rietveld refinement, conducted using TOPAS, was the single crystal structure of **1-*i*PrOH.** This model was refined with one global isotropic thermal parameter. A 6th order spherical harmonic correction of the intensities for preferred orientation was applied in the final stage of refinement. Rietveld refinement converged to R_{wp} of 0.10456, R_{wp}' = 0.18195 (R_{wp}' is the background subtracted R_{wp}); see Figure S5.

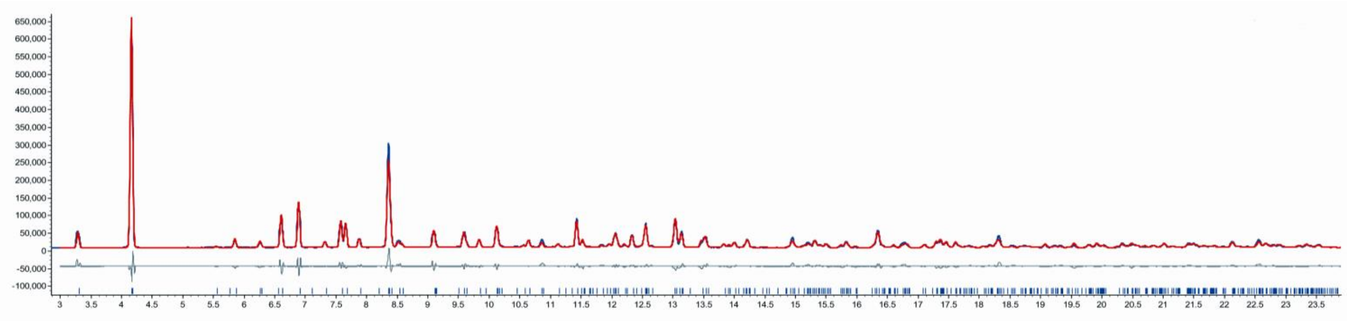

Figure S5. Observed (blue) and calculated (red) profiles and difference plot [I_{obs}-I_{calc}] (grey) of the Rietveld refinement of **1-*i*PrOH**. (2θ range: 3.0 – 24°; max. resolution 1.988 Å).

Solid-state reaction: 1-MeOH → 1-A^{HT} + 1-B^{HT} → 1-A^{HT} + 1-B^{HT} + 2 → 1-B^{HT} + 2 → 2

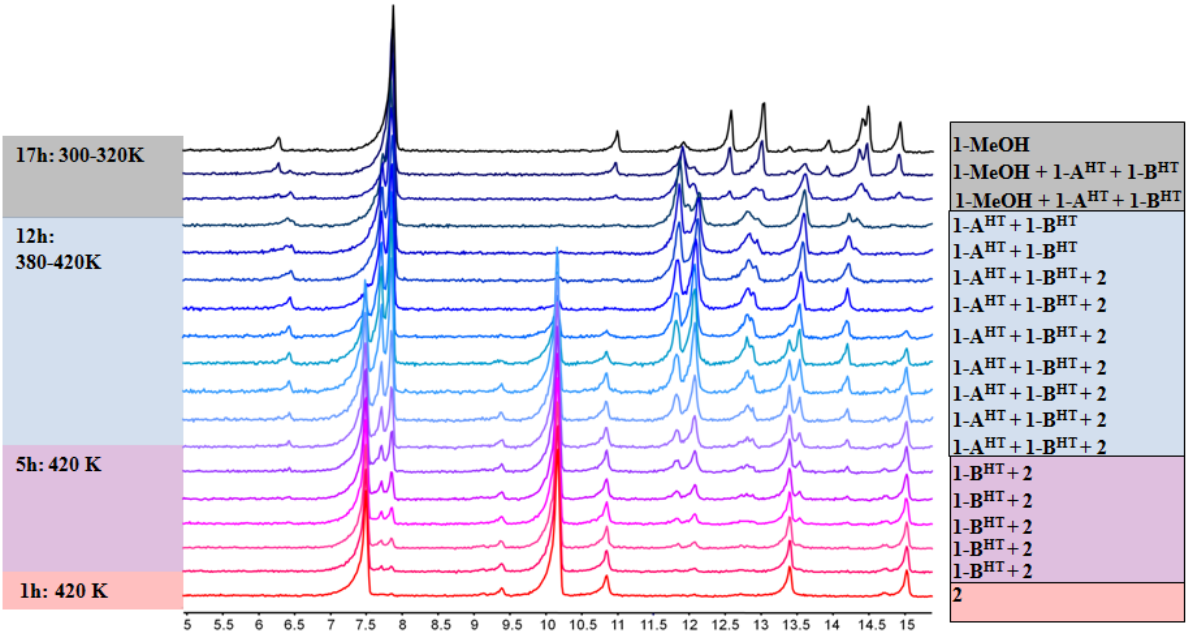

Figure S6. *In situ* X-ray powder diffraction study of heating of 1-MeOH, adapted from reference S2. The original report identified the two polymorphs of **1** as **1** and **1^{HT}**, rather than **1-A^{HT}** and **1-B^{HT}** used here.

Solid-state reaction transforming 1-EtOH → 1-A^{HT} + 1-B^{HT}

The gaps in the patterns that are not included in the fits result from the data collection method using the PSD detector on beamline I11. When this detector was first used by us at beamline I11, the data acquisition software did not provide an automated means to collect offset patterns and sum these to eliminate the gaps between modules. However, current data collection strategy and software can avoid this, as seen for the study of **1-*i*PrOH**.

Compound **3**, present as an impurity from the synthesis of **1-EtOH**, appears unchanged over the course of the loss of EtOH by **1-EtOH**. The gradual, but small, increase in percentage composition of **3** is attributed to some loss in crystallinity during the heating experiment. The contribution of amorphous material to the overall composition has not been determined quantitatively.

Starting material

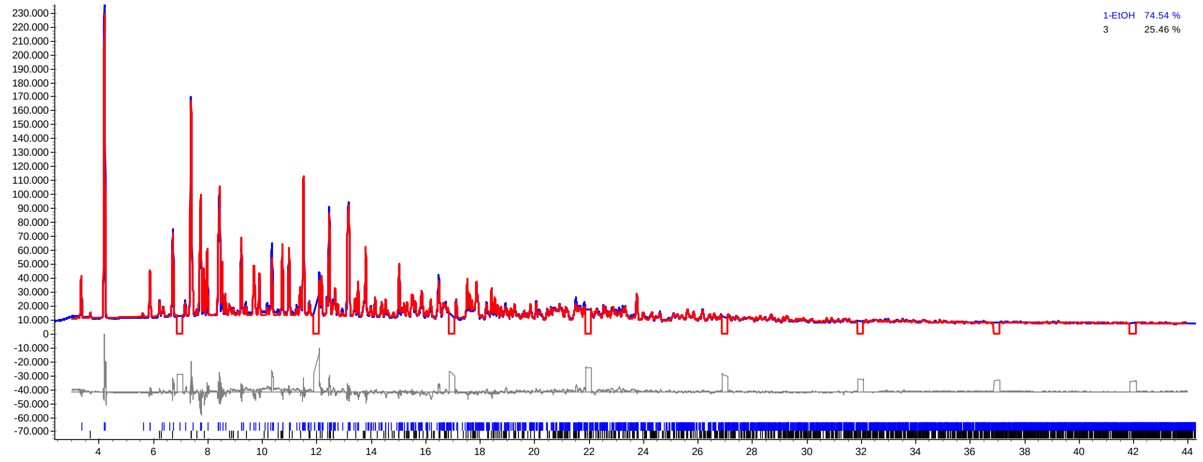

Figure S7. Observed (blue) and calculated (red) profiles and difference plot [(I_{obs} - I_{calcd})] (grey) of the Rietveld refinement for X-ray powder diffraction pattern ($3 \leq 2\theta \leq 44$ °, max. resolution 1.10Å) at 295 K (R_{wp} = 0.07695, R_{wp}' = 0.17843). Composition: **1-EtOH (**74.5(2) %) and **3** (25.5(1) %).

After 20 minutes at 340K

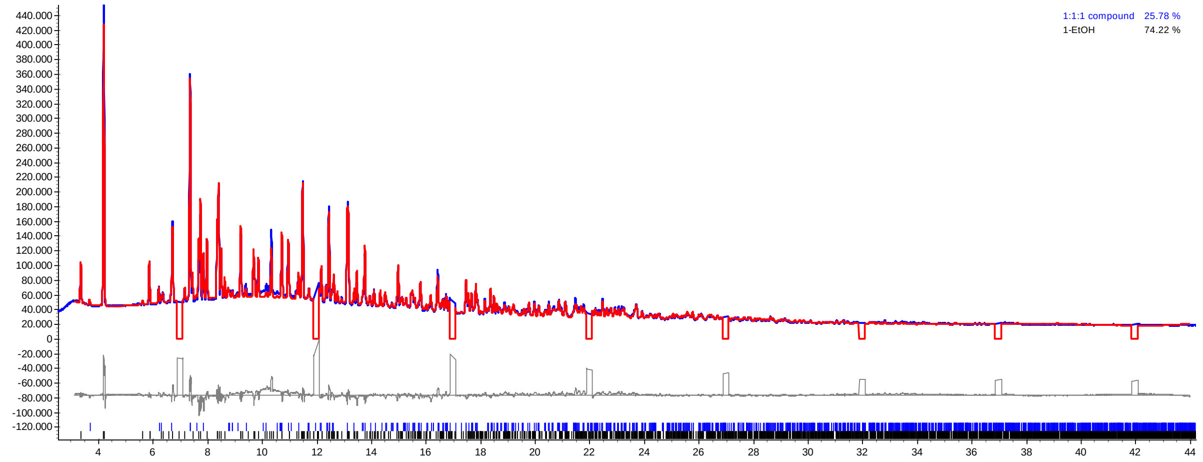

Figure S8. Observed (blue) and calculated (red) profiles and difference plot [(I_{obs} - I_{calcd})] (grey) of the Rietveld refinement for X-ray powder diffraction pattern ($3 \leq 2\theta \leq 44$ °, max. resolution 1.10 Å) at 340 K (R_{wp} = 0.05428, R_{wp}' =19.402). Composition: **1-EtOH (**74.2(2) %) and **3 (**25.8(2) %).

After 40 minutes at 340K

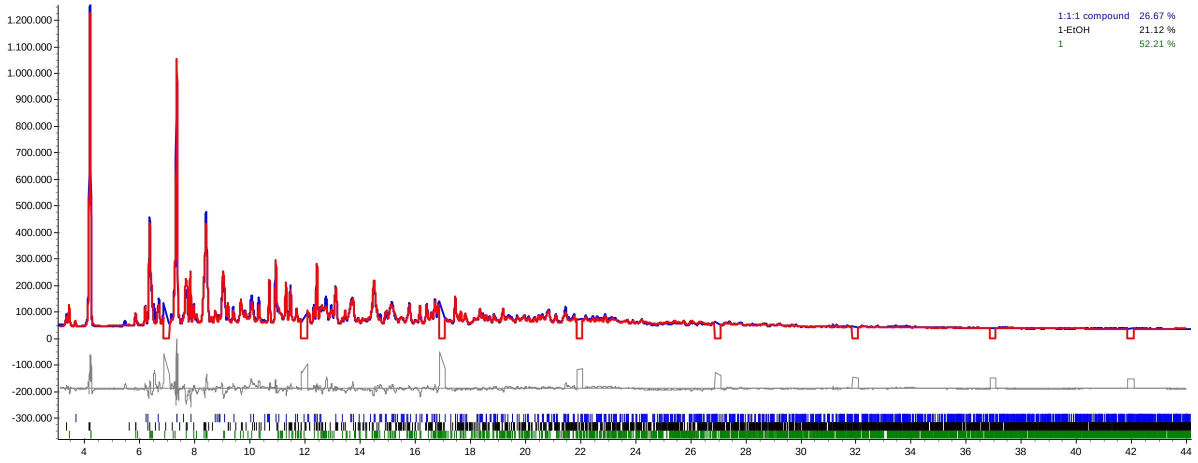

Figure S9. Observed (blue) and calculated (red) profiles and difference plot [(I_{obs} - I_{calcd})] (grey) of the Rietveld refinements for X-ray powder diffraction pattern ($3 \leq 2\theta \leq 44$ °, max. resolution 1.10 Å) at 340 K (R_{wp} = 0.08477, R_{wp}' = 0.20448). Composition: **1-EtOH (**21.1(4) %), of **3** (26.6(2) %), **1-A^{HT} (**41.0(2) %) and **1-B^{HT}** (10.1(2) %).

After 60 minutes at 340K

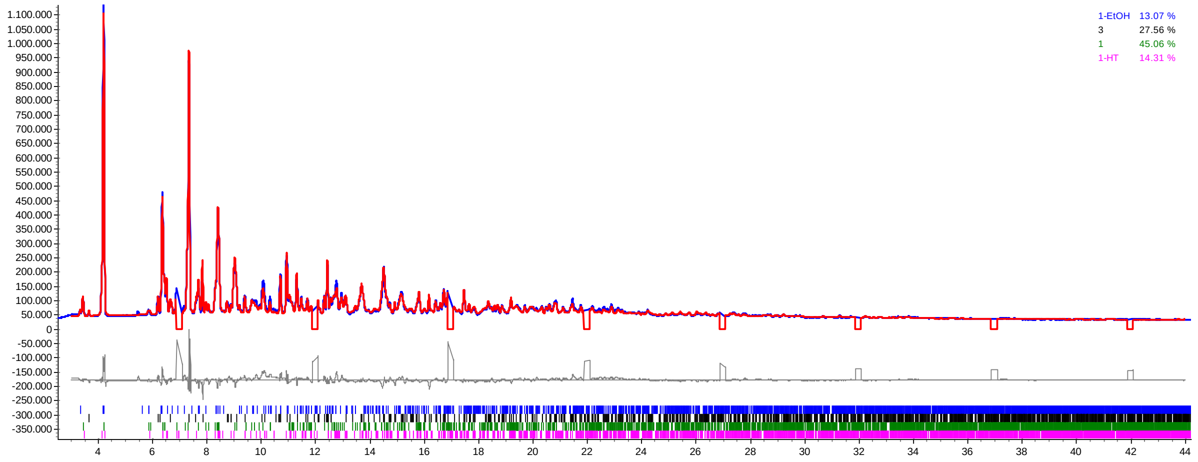

Figure S10. Observed (blue) and calculated (red) profiles and difference plot [(I_{obs} - I_{calcd})] (grey) of the Rietveld refinements for X-ray powder diffraction pattern ($3 \leq 2\theta \leq 44$ °, max. resolution 1.10Å) at 340 K (R_{wp} = 0.07257, R_{wp}' = 0.17635). Composition: **1-EtOH (**13.0(4) %), of **3** (27.6(3) %), **1-A^{HT} (**45.1(4) %) and **1-B^{HT}** (14.3(3) %).

After 80 minutes at 340K

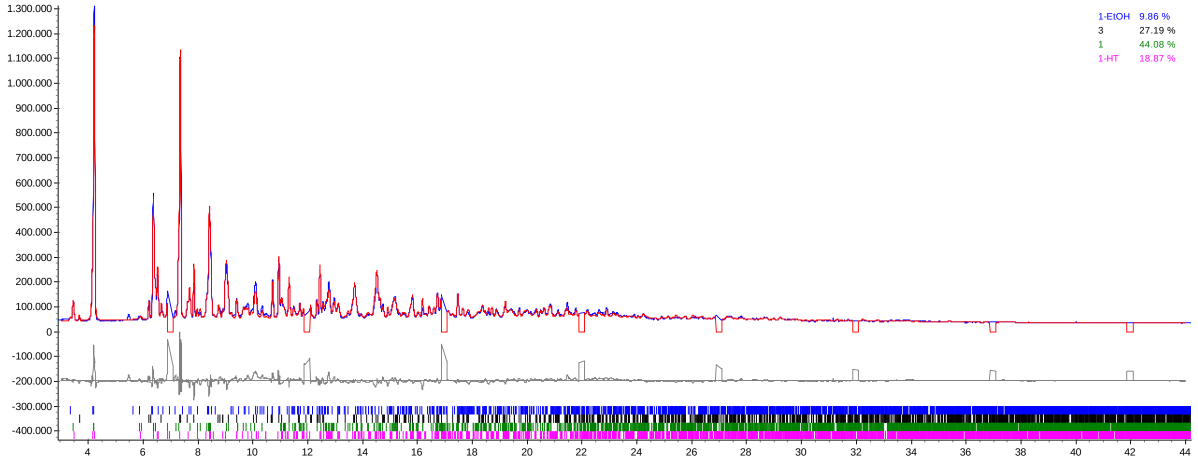

Figure S11. Observed (blue) and calculated (red) profiles and difference plot [(I_{obs} - I_{calcd})] (grey) of the Rietveld refinements for X-ray powder diffraction pattern ($3 \leq 2\theta \leq 44$ °, max. resolution 1.10Å) at 340 K (R_{wp} = 0.07752, R_{wp}' = 0.17908). Composition: **1-EtOH (**9.9(2) %), of **3** (27.2(1) %), **1-A^{HT} (**44.1(2) %) and **1-B^{HT}** (18.8(1) %).

After 100 minutes at 340K

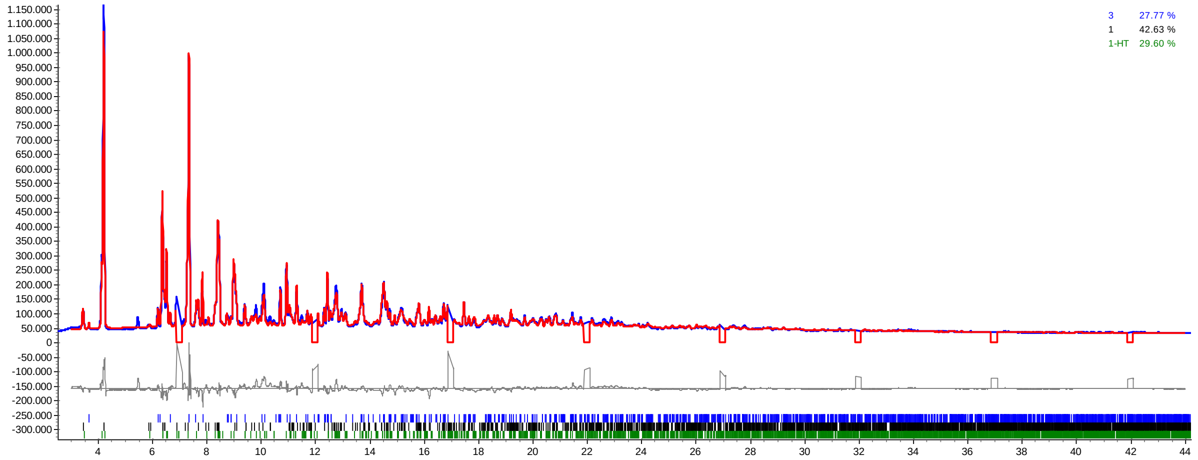

Figure S12. Observed (blue) and calculated (red) profiles and difference plot [(I_{obs} - I_{calcd})] (grey) of the Rietveld refinements for X-ray powder diffraction pattern (3 ≤ 2θ ≤ 44 °, max. resolution 1.10Å) at 340 K (R_{wp} = 0.07947, R_{wp}' = 0.19891). Composition: **3** (27.8(3) %), **1-A^{HT}** (42.6(4) %) and **1-B^{HT}** (29.6(4) %).

After 120 minutes at 340K

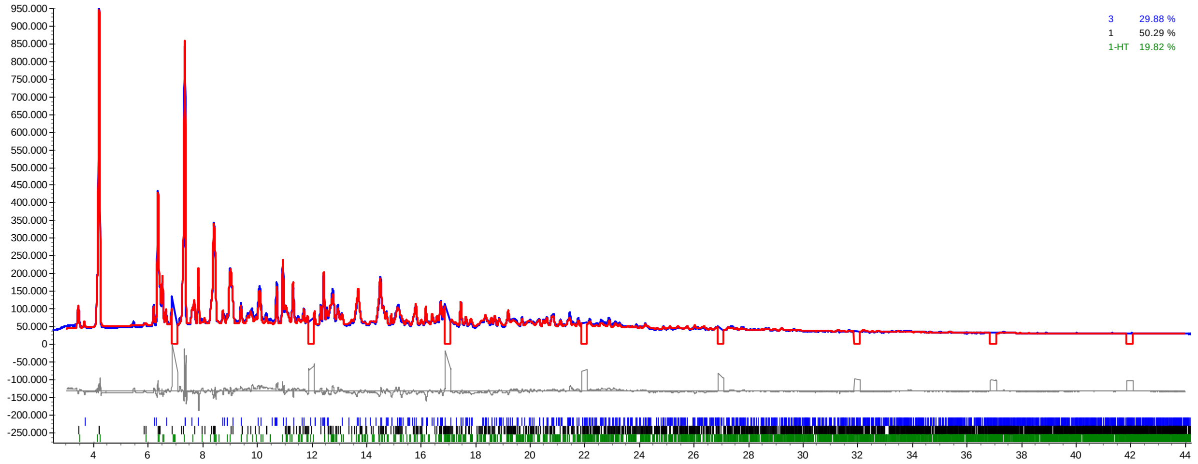

Figure S13. Observed (blue) and calculated (red) profiles and difference plot [(I_{obs} - I_{calcd})] (grey) of the Rietveld refinements for X-ray powder diffraction pattern (3 ≤ 2θ ≤ 44 °, max. resolution 1.10Å) at 340 K (R_{wp} = 0.06800, R_{wp}' = 0.18193). Composition: **3** (29.9(3) %), **1-A^{HT}** (50.3(4) %) and **1-B^{HT}** (19.8(3) %).

No further heating after 120 minutes at 340K – returned to room temperature

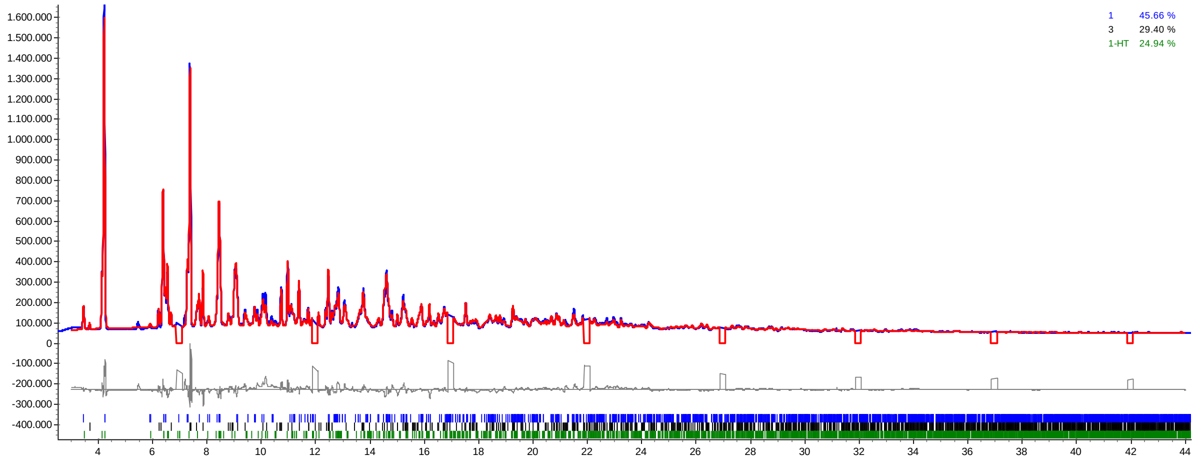

Figure S14. Observed (blue) and calculated (red) profiles and difference plot [(I_{obs} - I_{calcd})] (grey) of the Rietveld refinements for X-ray powder diffraction patterns (3 ≤ 2θ ≤ 44 °, max. resolution 1.10Å) at 295 K (R_{wp} = 0.07310, R_{wp}' = 0.17372). Composition: **3** (29.4(2) %), **1-A^{HT}** (45.6(3) %) and **1-B^{HT}** (24.9(3) %).

Solid-state reaction transforming 1-*i*PrOH → 1-B^{HT}

Starting material

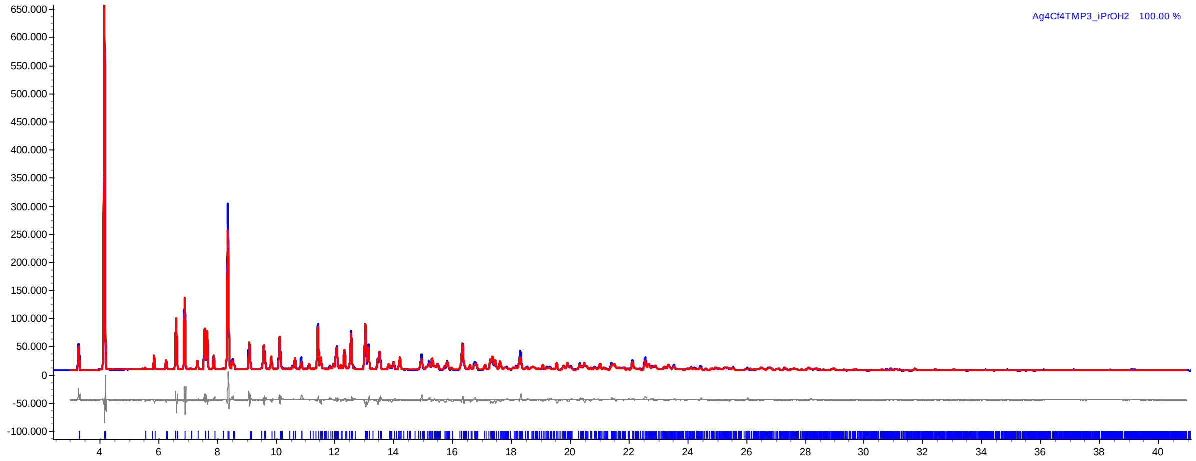

Figure S15. Observed (blue) and calculated (red) profiles and difference plot [(I_{obs} - I_{calcd})] (grey) of the Rietveld refinements for X-ray powder diffraction pattern ($3 \leq 2\theta \leq 41$ °, max. resolution 1.18Å) of **1-*i*PrOH** (100%) at 295 K (R_{wp} = 0.09758, R_{wp}' = 0.19581).

After 20 minutes heating at 373 K

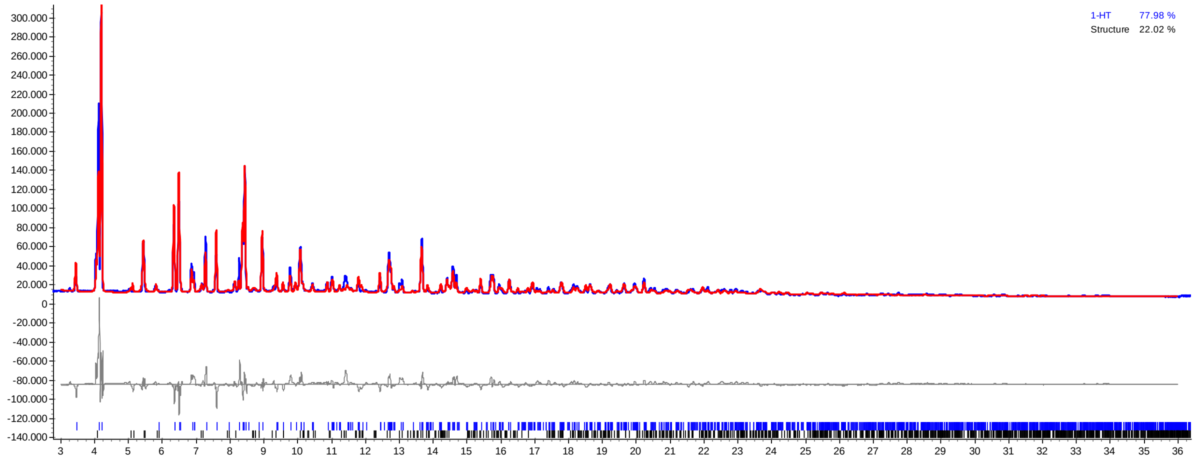

Figure S16. Observed (blue) and calculated (red) profiles and difference plot [(I_{obs} - I_{calcd})] (grey) of the Rietveld refinements for X-ray powder diffraction pattern ($3 \leq 2\theta \leq 36$ °, max. resolution 1.33Å) at 373 K (R_{wp} = 0.13226, $R_{wp'}$ = 0.32175). Composition: **1-B^{HT}** (78(1) %) and **2** (22(1) %).

No further heating – temperature returned to 295 K

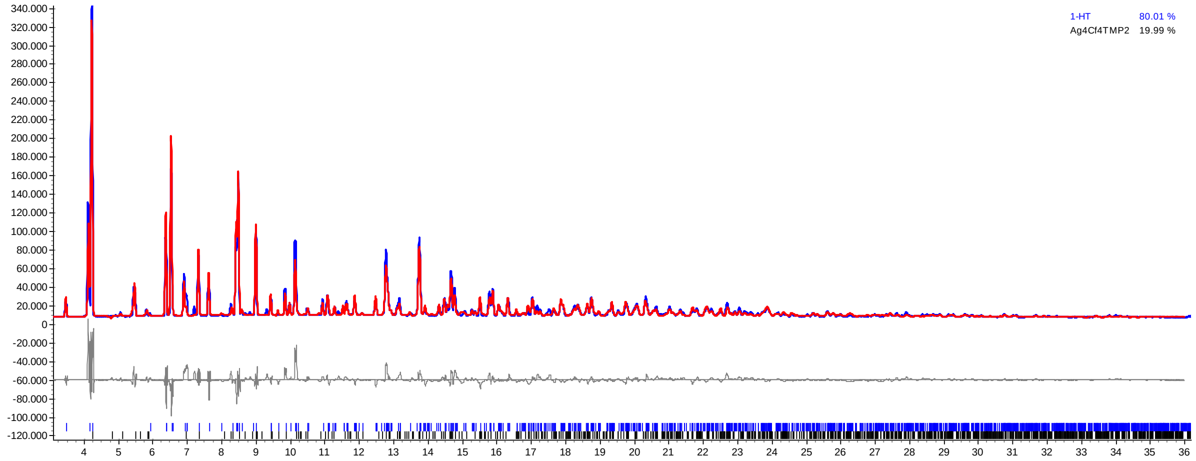

Figure S17. Observed (blue) and calculated (red) profiles and difference plot [(I_{obs} - I_{calcd})] (grey) of the Rietveld refinement for X-ray powder diffraction pattern (2 ≤ 2θ ≤ 36 °, max. resolution 1.33Å) at 295 K (R_{wp} = 0.14769, R_{wp}' = 0.30028). Composition: **1-B^{HT}** (80(1) %) and **2** (20(1) %).

AFM experiments: monitoring release of methanol by crystals of 1-MeOH

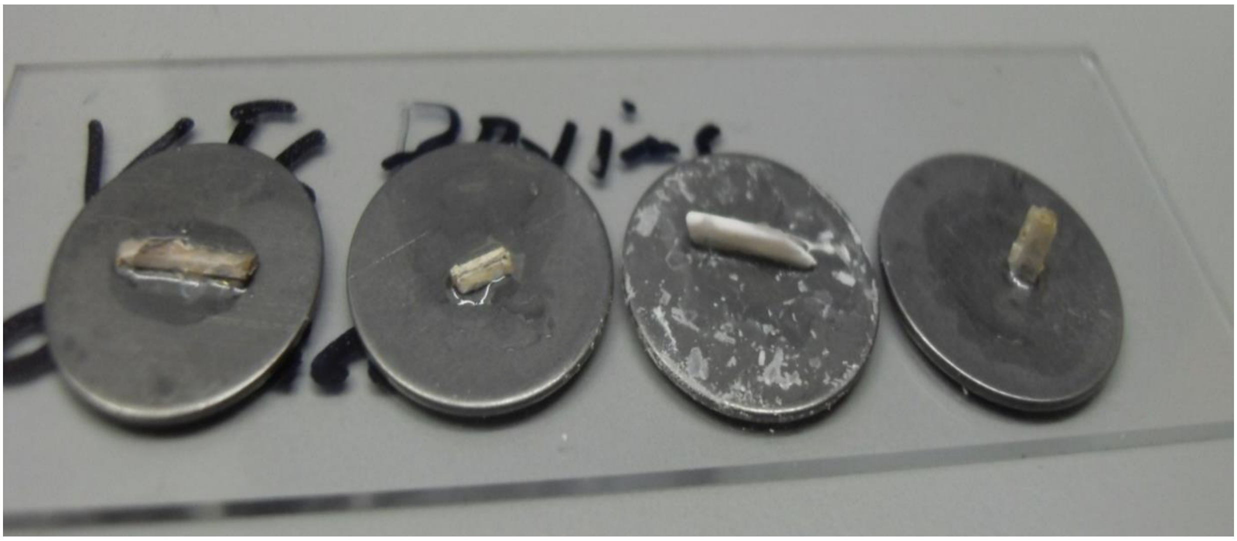

Figure S18. Image of the crystals **1-MeOH** glued to the disks in different orientations in order to monitor the different faces of the crystal to the MeOH release experiment by AFM.

Gas-phase Fourier-transform infra-red spectroscopy (FTIR)

Fourier transform infrared (FTIR) spectroscopic experiments were conducted using a double-walled 10 cm glass IR absorption cell fitted with either KCl or KBr windows and a suspended sample container (Figure S19) as previously described.[S8] To obtain the calibration line for methanol, ethanol and isopropanol vapours, gas-phase IR spectra in the region of 400-4000 cm^{-1} were acquired using a FTIR spectrometer (Perkin- Elmer Paragon 1000, resolution 1 cm^{-1}, no apodization). The spectrometer was operated in the single-beam mode, that is, sample and background (empty cell) spectra were recorded separately. The calibration for the FTIR experiment was performed by introducing a known amount of dry ROH (ROH = MeOH, EtOH and ^{i}PrOH), which was previously de-gassed by freeze-pump-thaw methods, into an empty gas cell (evacuated under vacuum) and acquiring IR spectra at 22 ºC (range of pressures used for calibration: 5-30 Torr). The absorbance is given as lg(I_0/I). The area under the methanol C–O stretching absorption band was integrated from 950 to 1100 cm^{-1} after background subtraction and baseline correction to determine the partial pressure of methanol (Figures S20 and S21). For ethanol and isopropanol the areas under the C–O stretching and C−H bending absorption bands, which overlap, was integrated from 950 to 1175 cm^{-1} and 900 to 1000 cm^{-1}, respectively, after background subtraction and baseline correction (Figures S22-S25).

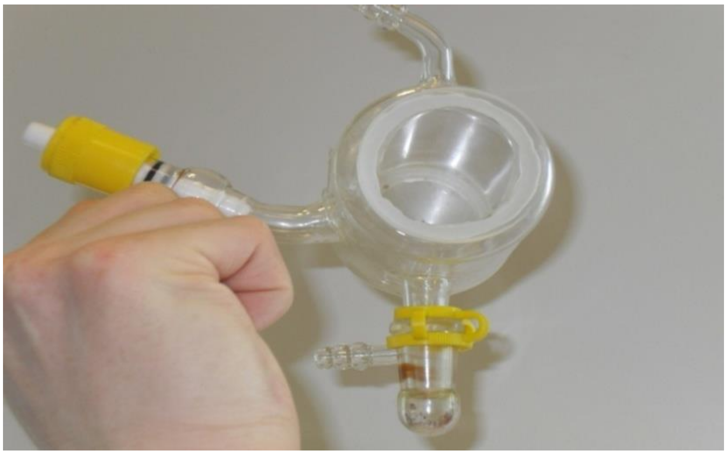

Figure S19. Double-walled 10 cm glass IR absorption cell fitted with KCl windows.

Gas phase methanol FTIR

Dry methanol at pressures of 5, 10, 15, 20 and 30 Torr was used to determine the calibration line. The integrated absorbance was measured by integrating the area of the peaks in the region of 950–1100 cm^{-1} (C−O stretching band, Figure S20)

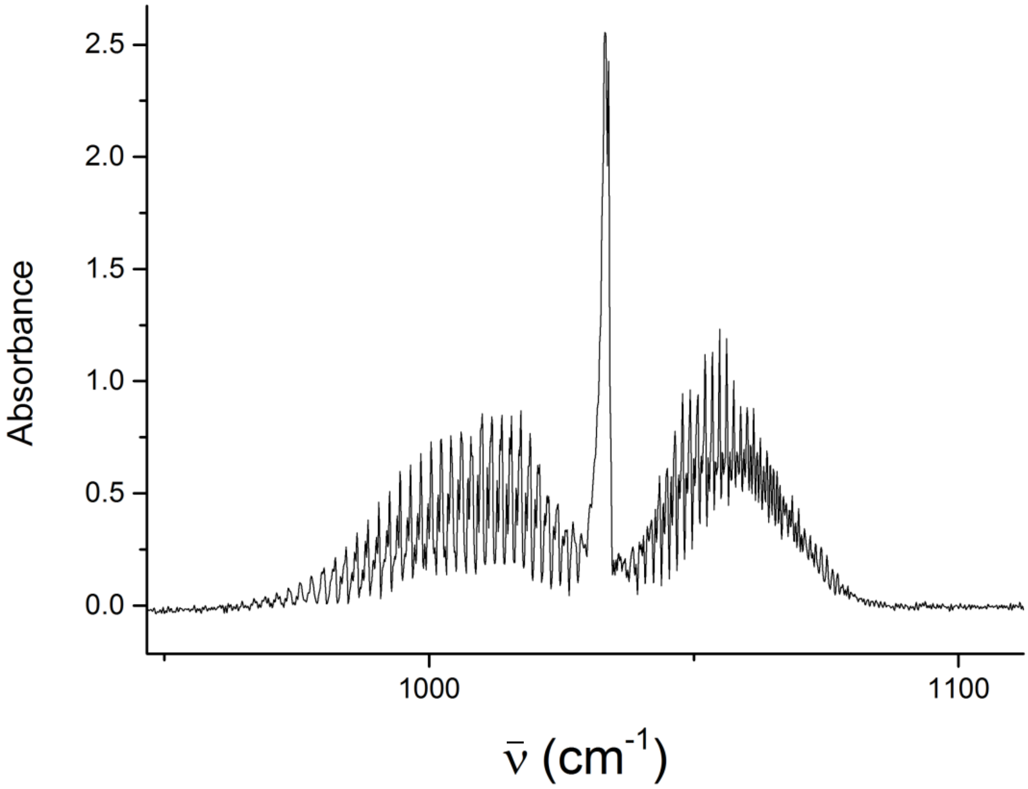

Figure S20. Methanol C−O stretching band in the gas phase including P and R branches of the rotational fine structure at a partial pressure of 30 Torr.

One hundred spectra were measured for each pressure. The final calibration line has R^2 = 0.9999 corresponding to Integrated Absorbance (cm^{-1}) = 4.1826 × Pressure (bar) (Figure S21).

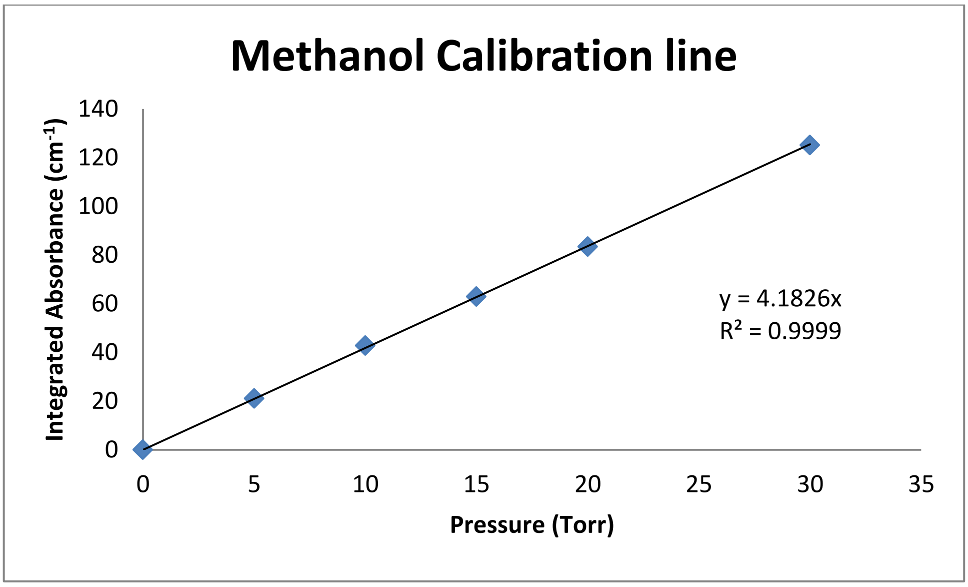

Figure S21. Methanol pressure calibration line.

Table S2. Data corresponding to the ethanol calibration line in Figure S21

**Pressure (Torr)**	**Integrated absorbance (cm^{-1})**
0	0
5	21.063
10	42.773
15	62.860
20	83.448
30	125.214

Gas phase ethanol FTIR

Dry ethanol at pressures at 5, 10, 15, 20 and 30 Torr was used to determine the calibration line. The integrated absorbance was measured by integrating the area of the peaks in the region of 950–1175 cm^{-1} (C–O stretching and C–H bending bands, Figure S22)

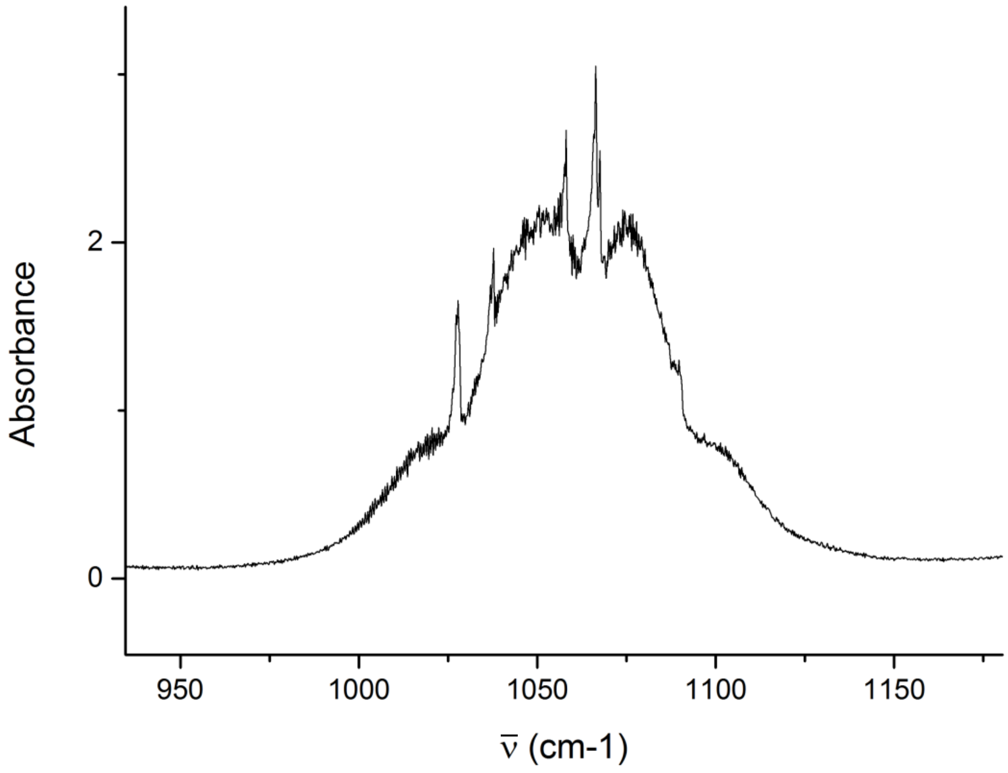

Figure S22. Ethanol C−O stretching and C–H bending bands in gas phase at a partial pressure of 30 Torr.

One hundred spectra were measured at each pressure. The final calibration line has R^2 = 0.9998 corresponding to Integrate Absorbance (cm^{-1}) = 4.8512 × Pressure (Torr) (Figure S23).

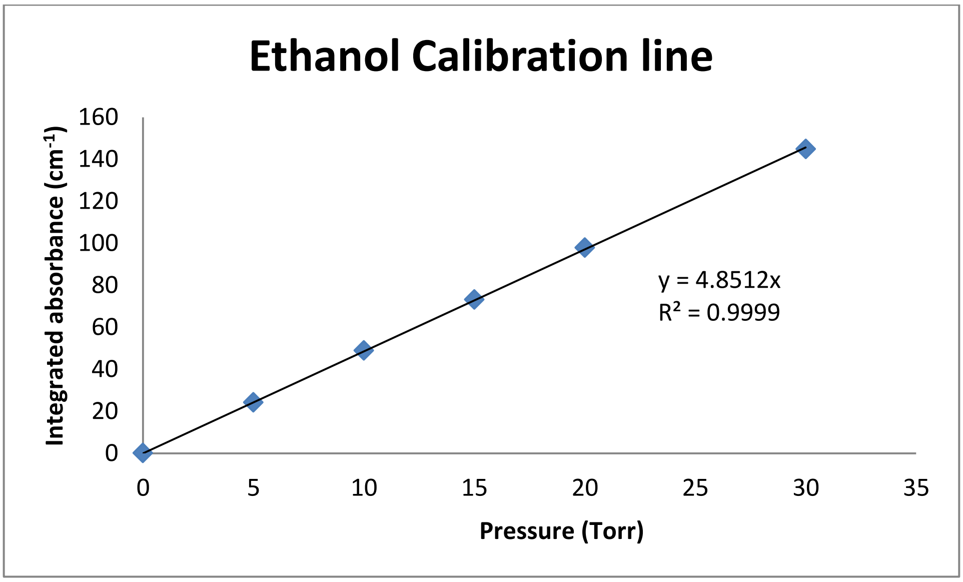

Figure S23. Ethanol pressure calibration line

Table S3. Data corresponding to the ethanol calibration line

Pressure (Torr)	Integrated absorbance (cm^{-1})
0	0
5	24.163
10	48.810
15	73.042
20	97.811
30	144.792

Gas phase Isopropanol FTIR

Dry isopropanol at pressures of 5, 10, 15, 20 and 25 Torr was used to determine the calibration line. The integrated absorbance was measured by integrating the area of the peaks in the region of 900–1000 cm^{-1} (C−O stretching and C–H bending bands, Figure S24).

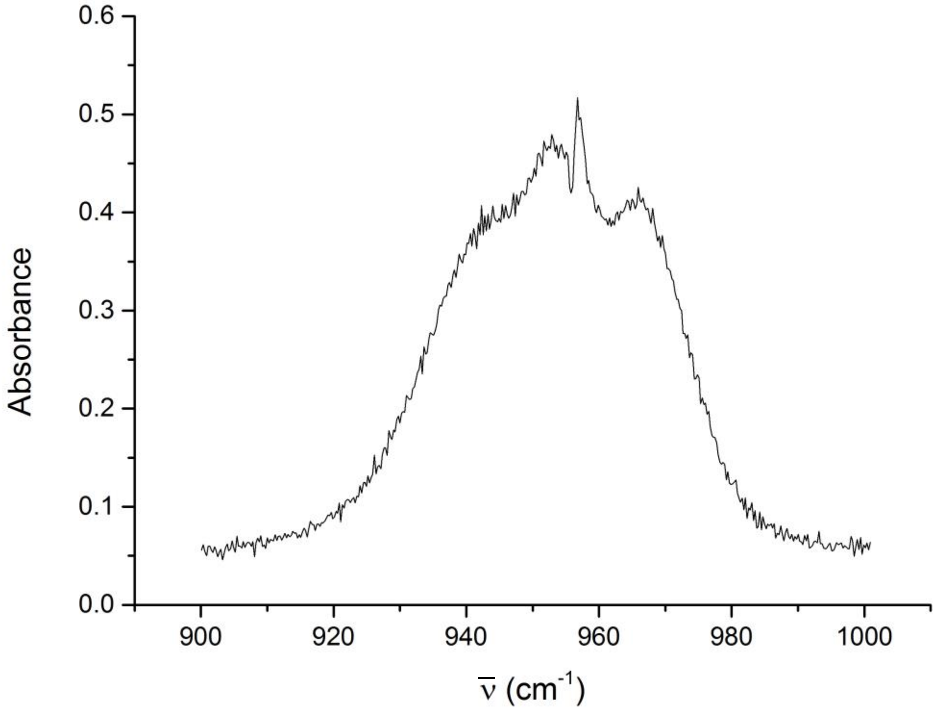

Figure S24. Isopropanol C−O stretching and C–H bending bands in gas phase at a partial pressure of 25 Torr.

One hundred spectra were measured at each pressure. The final calibration line has R^2 = 0.9972 corresponding to Integrated Absorbance (cm^{-1}) = 0.643 × Pressure (Torr) (Figure S25).

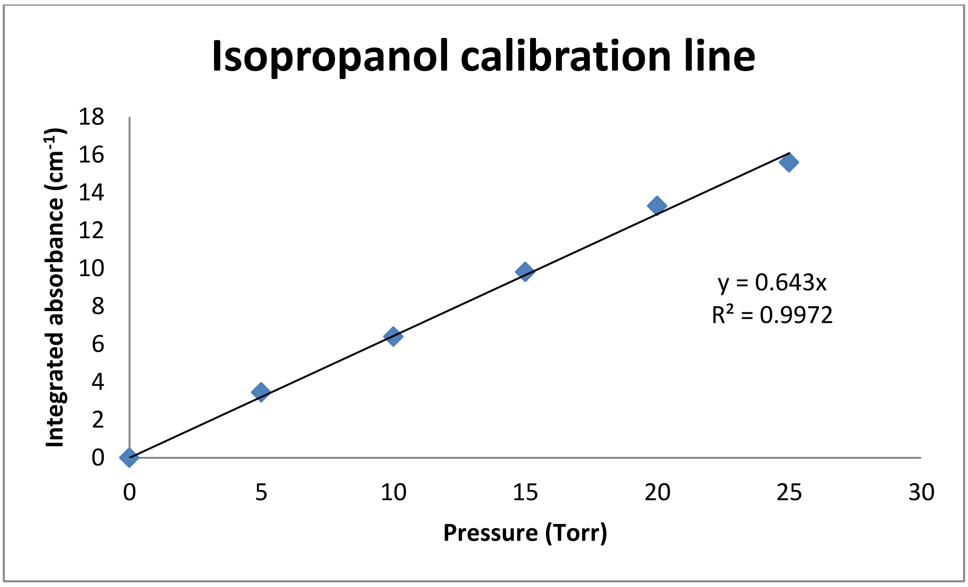

Figure S25. Isopropanol pressure calibration line

Table S4. Data corresponding to the isopropanol calibration line

Pressure (Torr)	Integrated absorbance (cm^{-1})
0	0
5	3.448
10	6.396
15	9.810
20	13.292
25	15.599

The equilibria linking the crystalline solids **1** and **1-ROH** and the vapour ROH are:

$$[Ag_4(O_2C(CF_2)_2CF_3)_4(TMP)_3(ROH)_2] (s) \rightleftharpoons [Ag_4(O_2C(CF_2)_2CF_3)_4(TMP)_3] (s) + 2 ROH (g)$$

(1-ROH) **(1)**

In order to calculate the equilibrium constant a series of equations were used. Equation (1) defines the equilibrium constant, where a_1 is the activity of compound **1**; $f_{\mathbf{ROH}}$ is the fugacity of the alcohol released and, $a_{\mathbf{1\text{-}ROH}}$ is the activity of the alcohol-containing coordination polymer **1-ROH**. Equation (2) defines the equilibrium constant in pressure terms, where $p_{\mathbf{ROH}}$ is the equilibrium pressure of the alcohol and $\varphi_{\mathbf{ROH}}$ is the fugacity coefficient, which is defined in equation (3). As the final alcohol pressures were very low (see Table 2), it can be supposed that the alcohols behave as perfect gases, so the fugacity coefficient is unity. In order to study the activity of the coordination polymers, an independent methanol release experiment was done, doubling the amount of initial **1-MeOH** coordination polymer. The equilibrium pressure ($p_{\mathbf{MeOH}}$ = 0.00753(6) bar) is similar to the experiment done with only 50 mg of starting **1-MeOH** coordination polymer. It can be concluded that the equilibrium pressure is independent of the amount of starting material. As a consequence, the activity coefficients of the two solid components of the reaction can be assumed to be unity.

$$K_{eq} = a_1 \times (f_{\mathbf{ROH}}/p^o)^2 / (a_{\mathbf{1\text{-}ROH}}) \quad (1)$$

$$K_p = (f_{\mathbf{ROH}}/p^o)^2 = (\varphi_{\mathbf{ROH}} \times p_{\mathbf{ROH}}/p^o)^2 \quad (2)$$

$$\varphi = \lim_{p \to 0}(f/p) \quad (3)$$

The equilibrium constant and the Gibbs free energy for each of the three equilibria are provided in Table 4.

TGA and DSC traces

a)

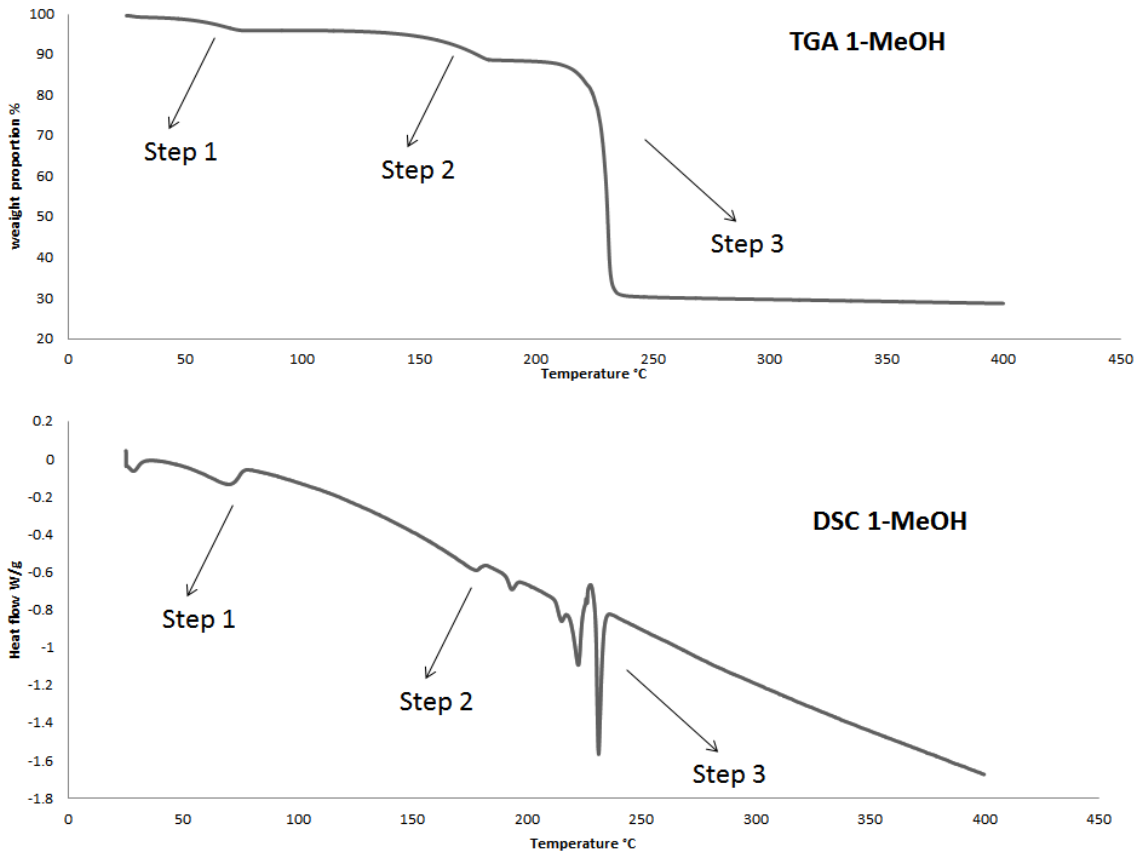

b)

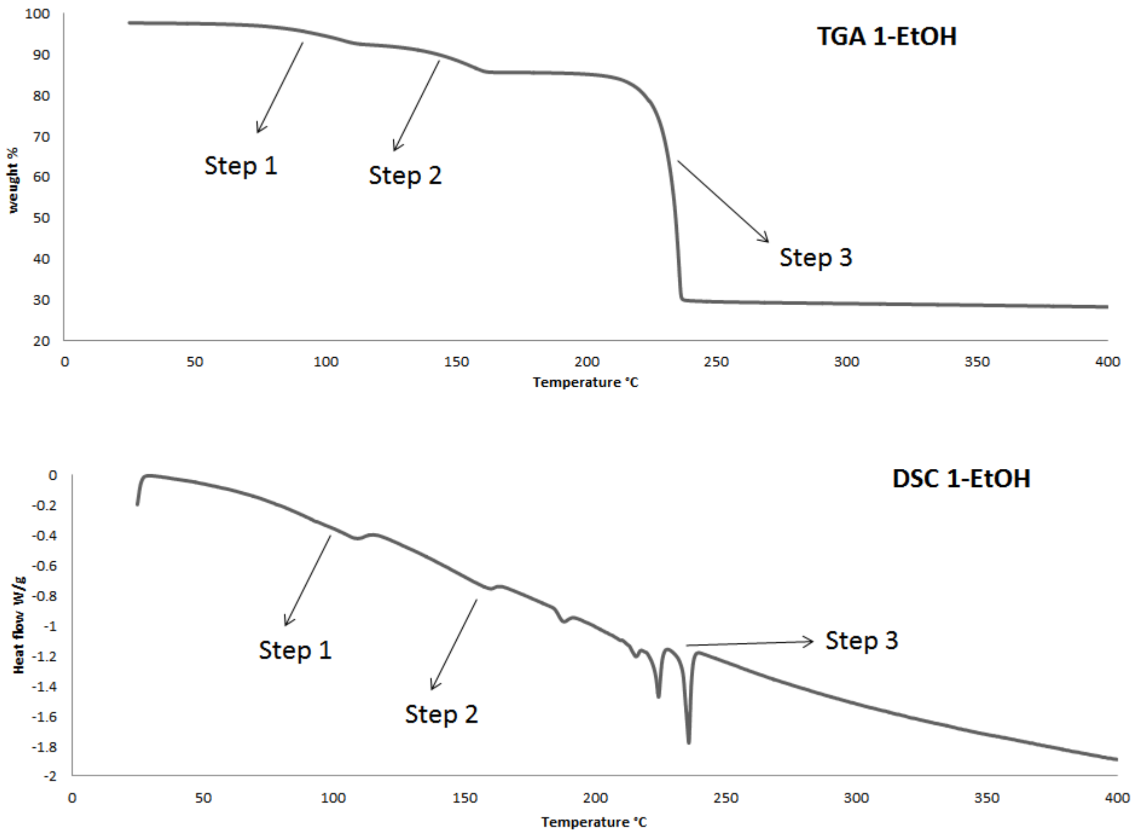

c)

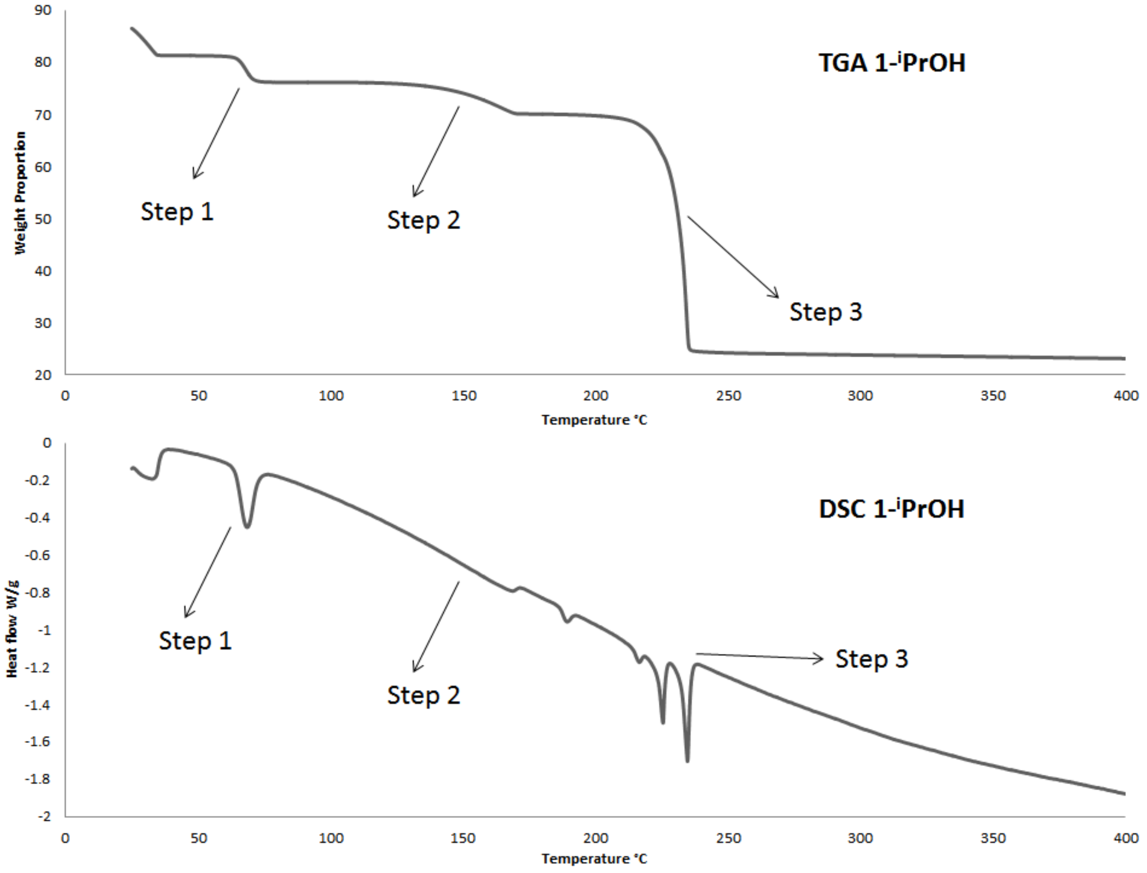

Figure S26. TGA-DSC analysis for (a) **1-MeOH**, (b) **1-EtOH** and (c) **1-*i*PrOH**. See Table 1 for numerical data and associated text for discussion of these experimental data.

References

S1. *Rietveld refinement*: H. M. Rietveld, *J. Appl. Crystallogr.* **1969**, *2*, 65-71.
S2. I. J. Vitoríca-Yrezábal, G. Mínguez Espallargas, J. Soleimannejad, A. J. Florence, A. J. Fletcher, L. Brammer, *Chem. Sci.* **2013**, *4*, 696-708.
S3. *TOPAS*: A. A. Coelho, *TOPAS Academic, Version 4.1*, 2007; see http://www.topas-academic.net.
S4. *Pawley refinement*: G. S. Pawley, *J. Appl. Crystallogr.* **1981**, *14*, 357-361.
S5. *ID31 (ESRF)*: A. N. Fitch, *J. Res. Natl. Inst. Stand. Technol*. **2004**, *109*, 133.
S6. *I11 (DLS)*: S. P. Thompson, J. E. Parker, J. Potter, T. P. Hill, A. Birt, T. M. Cobb, F. Yuan and C. C. Tang, *Rev. Sci. Instrum.* **2009**, *80*, 075107.
S7. *I11 (PSD detector), DLS*: S. P. Thompson, J. E. Parker, J. Marchal, J. Potter, A. Birt, F. Yuan, R. D. Fearn, A. R. Lennie, S. R. Street, and C. C. Tang, *J. Synchrotron Rad*. **2011**, *18*, 637.
S8. G. Mínguez Espallargas, M. Hippler, A. J. Florence, P. Fernandes, J. van de Streek, M. Brunelli, W. I. F. David, L. Brammer, *J. Am. Chem Soc*. **2007**, *129*, 15606-15614.
